# Supplementary material for: Brain health measurement: a scoping review
Source: BMJ Open. 2024 Feb 10;14(2):e080334. doi: 10.1136/bmjopen-2023-080334 (PMC10862273; doi:10.1136/bmjopen-2023-080334)
Supplement: Supplementary data [file bmjopen-2023-080334supp001.pdf]

# Appendices and supplementary material

## APPENDIX I: SEARCH STRATEGY

("brain health"(TextWord) AND Measurement terms (All Fields))
OR
((("Cerebrospinal fluid" OR "diagnostic imaging" OR anisotropy OR apolipoprotein OR apolipoproteins OR "arterial spin labelling" OR "brain derived neurotrophic factor" OR biomarker OR biomarkers OR "blood oxygen level dependent" OR "brain function" OR "brain imaging" OR "brain volume" OR "brain tissue volume" OR "cognitive assessment" OR "cognitive interview" OR "cognitive interviews" OR "DNA methylation" OR "genome wide association study" OR GWAS OR "gray matter" OR "grip strength" OR "hand strength" OR "hippocampal volume" OR "magnetic resonance imaging" OR MRI OR "magnetic resonance spectroscopy" OR "mental health statistics" OR "mental status and dementia tests" OR "mini mental state exam" OR "mini mental state examination" OR MMSE OR "motor control" OR "neurocognitive function" OR neuroimaging OR "neuropsychological tests" OR "neuropsychological testing" OR "non verbal reasoning" OR "nonverbal reasoning" OR "nutritional status" OR "physical function" OR "positron emission tomography" OR proteomic OR proteomics OR "psychological tests" OR "psychological testing" OR "self report" OR "short sleep duration" OR "surveys and questionnaires" OR "computed tomography" OR "voxel based morphometry" OR "white matter hyperintensities")) AND ((brain[Title] OR "white matter"[Title] OR Brain[MeSH Terms]) AND (atroph*[Title] OR Alzheimer*[Title] OR cogniti*[Title] OR dementia*[Title] OR "executive function"[Title] OR hippocamp*[Title] OR "mild cognitive impairment"[Title] OR memory[Title] OR "mental disorder"[Title] OR "mental health"[Title] OR neurocognitive[Title] OR neurodegeneration[Title] OR "postoperative cognitive"[Title] OR stroke*[Title] OR "vascular cognitive"[Title]) AND (health[Title] OR healthy[Title] OR longevity[Title] OR "protective factor*"[Title] OR "quality of life"[Title] OR psycholog*[Title] OR resilience[Title] OR well-being[Title] OR health[MeSH Terms] OR outcome assessment health care[MeSH Terms] OR quality of life[MeSH Terms] OR psychological well-being[MeSH Terms]))) OR (("brain health"[Text Word]) AND (("Cerebrospinal fluid" OR "diagnostic imaging" OR anisotropy OR apolipoprotein OR apolipoproteins OR "arterial spin labelling" OR "brain derived neurotrophic factor" OR biomarker OR biomarkers OR "blood oxygen level dependent" OR "brain function" OR "brain imaging" OR "brain volume" OR "brain tissue volume" OR "cognitive assessment" OR "cognitive interview" OR "cognitive interviews" OR "DNA methylation" OR "genome wide association study" OR GWAS OR "gray matter" OR "grip strength" OR "hand strength" OR "hippocampal volume" OR "magnetic resonance imaging" OR MRI OR "magnetic resonance spectroscopy" OR "mental health statistics" OR "mental status and dementia tests" OR "mini mental state exam" OR "mini mental state examination" OR MMSE OR "motor control" OR "neurocognitive function" OR neuroimaging OR "neuropsychological tests" OR "neuropsychological testing" OR "non verbal reasoning" OR "nonverbal reasoning" OR "nutritional status" OR "physical function" OR "positron emission tomography" OR proteomic OR proteomics OR "psychological tests" OR "psychological testing" OR "self report" OR "short sleep duration" OR "surveys and questionnaires" OR "computed tomography" OR "voxel based morphometry" OR "white matter hyperintensities")))



Lee A, *et al. BMJ Open* 2024; 14:e080334. doi: 10.1136/bmjopen-2023-080334

Search limits: English language, Human

## APPENDIX II: PRISMA DIAGRAM

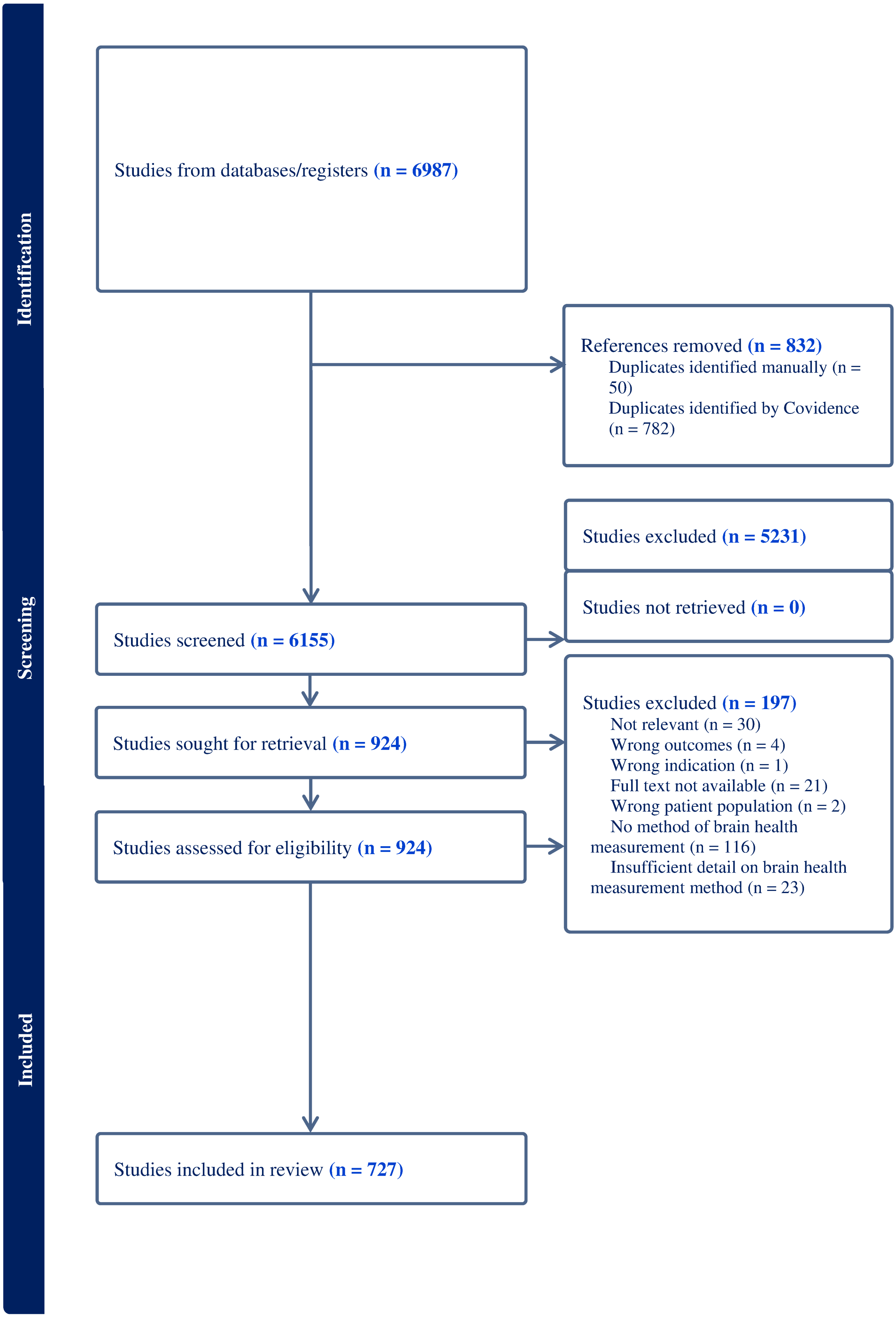




Lee A, *et al. BMJ Open* 2024; 14:e080334. doi: 10.1136/bmjopen-2023-080334

## APPENDIX III: DATA EXTRACTION INSTRUMENT

*All numbered items were presented as checkboxes for reviewers to tick for all that applied for each study.*

### Study characteristics

Author last name [whitespace]

Year of publication[whitespace]

Type of study

1. Randomised controlled trial
2. Cohort study
3. Systematic review/meta-analysis
4. Narrative review
5. Case series
6. Other

Any specific definition of brain health listed in paper? (optional) [whitespace]

### Methods of brain health measurement

### Imaging

Structural MRI

1. Total/whole brain volume
2. Total/whole brain gray matter volume
3. Total/whole brain white matter volume
4. Hippocampal volume
5. Gray matter volume in specific region(s)
6. White matter volume in specific region(s)
7. Cerebrospinal fluid (CSF) volume in specific region(s)
8. White matter hyperintensities (WMHs)
9. Cerebral microbleeds (CMBs)
10. Cortical thickness
11. White matter lesion volume

Functional MRI

1. Resting state functional connectivity
2. Task based functional connectivity
3. Cerebral blood flow (arterial spin labelling/BOLD responses)
4. Cerebral blood volume
5. Cerebral oxygen consumption
6. Cerebral metabolic rate of oxygen (CMRO2)

Diffusion MRI

1. Mean diffusivity
2. Axial diffusivity
3. Radial diffusivity
4. Fractional anisotropy
5. Fibre bundle lengths

Compound imaging

1. Brain age estimations (image based)
2. Brain age gap calculations
3. Brain atrophy and lesion index (BALI)
4. Brain health quotients (BHQ)

PET scans/ Spectroscopy/Misc

1. Amyloid load/status
2. Lactate
3. N-acetylaspartate
4. Glutamate
5. Glutamine
6. Magnetoencephalography (MEG) - functional connectivity

Other imaging parameters not previously specified[whitespace]

**Genetics/blood/CSF/other**

Genetics

1. ApoE4 allele/gene variation

Blood/CSF biomarkers

1. BDNF (serum/blood)
2. BDNF (CSF)
3. Tau (blood)
4. Tau (CSF)
5. Abeta42 or 40 (blood)
6. Abeta 42 or 40 (CSF)
7. IL8 (blood)
8. IL8 (CSF)
9. ACE angiotensin converting enzyme (blood)

EEG

1. Mindreader
2. EEG based brain age
3. EEG unspecified

Clinical

1. Hand grip strength
2. Clinical diagnosis of alzheimer's disease/dementia
3. Oculometric test
4. Duplex ultrasonography of carotids
5. Transcranial doppler ultrasound
6. Transcranial magnetic stimulation (TMS)

Mental health

1. Hospital anxiety and depression (HADS) scales
2. Centre for epidemiologic studies depression scale (CES-D)
3. Beck anxiety inventory
4. Beck depression inventory-ii
5. Pittsburgh Sleep Quality index
6. Mayo clinic fluctuations scale
7. Quality of life scale
8. Zung self rating depression scale
9. Self reported mental health history
10. Apathy scale

Combination scores

1. Brain health score (lifestyle based)
2. Brain health test (BHT)
3. Lifestyle for brain health (LIBRA) index
4. Resilience index

Any other clinical/mental health/combo scores/genetic measures not previously reported[whitespace]

**Cognitive tests**

Cognitive tests (A-Z)

1. Mini-mental status examination (MMSE)
2. Montreal cognitive assessment (MoCA)
3. Activities specific balance confidence scale
4. Adulthood cognitive activity questionnaire
5. Alzheimer's disease assessment cale (ADAS-cog)
6. Animal naming
7. Apathy inventory
8. Attention network test (ANT)
9. Auditory consonant trigrams
10. Awareness of social inference test (TASIT-R)
11. Behaviour rating inventory of executive function adult version (BRIEF-A)
12. Behavioural assessment of dysexecutive syndrome (BADS)
13. Bell cancellation test
14. Benson Figure Recall
15. Benton Visual Retention Test (BVRT)



Lee A, *et al. BMJ Open* 2024; 14:e080334. doi: 10.1136/bmjopen-2023-080334

16. Boston diagnostic aphasia exam
17. Boston naming test (BNT)
18. Brief cognitive ability measure (B-CAM)
19. Brief visuospatial memory test-revised (BVMT)
20. Buschke selective reminding test
21. California verbal learning test (CVLT-II)
22. Cambridge Cognition Paired Associates Learning
23. CANTAB intra extra dimensional set shift (IED)
24. Category fluency
25. CERAD word list delayed recall and memory battery
26. Clinical dementia rating (CDR)
27. Clock drawing test (CDT)
28. CNS vital signs
29. Cogniciti's brain health assessment (BHA)
30. Cogstate brief battery (CBB)
31. Cogstate neurocognitive battery
32. Cohen's relational memory
33. Color trails test
34. Color word interference test (CWIT)
35. COMPASS-ND neuropsychological battery
36. Continuous paired associative learning (CPAL) task
37. Controlled oral word association test
38. Corsi block test
39. Delis-Kaplan executive functioning (D-KEFS)
40. Digit span
41. Digit symbol substitution test (DSST)
42. Direct assessment of functional status revised (DAFS-R)
43. DKEFS Color Word Intereference
44. Edinburgh Handedness Inventory (EHI)
45. Everyday cognition scale (ECog)
46. Face name association test
47. Flanker NIH Toolbox
48. Frontal assessment battery (FAB)
49. Frontal systems behaviour scale (FrSBe)
50. Geriatric anxiety inventory
51. Gerontology functional assessment tool (NCGG-FAT)
52. Grooved pegboard (PEGS)
53. Hasegawa's dementia scale-revised (HDS-R)
54. Hooper visual organisation test
55. Hopkins verbal learning test (HVLT)
56. Identical pairs version
57. Illness intrusiveness rating scale (IIRS)
58. Inspection time task
59. Iowa Gambling Task
60. IQ test
61. Letter fluency
62. Letter number sequencing
63. Lifetime of experiences questionnaire (LEQ)
64. Logical memory

65. MATRICS consensus cognitive battery (MCCB)
66. Mattis dementia rating scale (DRS)
67. Maryo clinic study of ageinig (MCSA)
68. Memory tool box task
69. Memtrax memory test
70. MiniCog
71. Mnemonic similarity task (MST)
72. ModBent
73. [Modified] california verbal learning test
74. [Modified] Rey Auditory verbal learning test (RAVLT)
75. Moray House Test No.12
76. MSCEIT managing emotions
77. N- back working memory task
78. NAB mazes
79. National adult reading test
80. National institutes of health (NIH) Toolbox-cognitive battery
81. Neuropsychological assessment scales
82. Number letter computer task
83. Number symbol coding task
84. Pfeffer questionnaire
85. Philadelphia verbal learning test (PVLT)
86. Pittsburgh compound B
87. Positive and negative affect schedule (PANAS)
88. Preclinical Alzheimer's cognitive composite 5 (PACC5)
89. Reaction time tasks
90. Repeatable battery for the assessment of neuropsychological status (RBANS)
91. Rey-Osterrieth Complex Figure test (CFT)
92. RI-48 Cued recall test
93. Set switching
94. Shopping list memory task
95. Short portable mental status questionnaire (SPMSQ)
96. Spatial working memory
97. Stroop color-word test (SCWT)
98. Subjective cognitive decline-questionnaire (SCD-Q)
99. Sustained attention to response task (SART)
100. Symbol digit modalities test (SDMT)
101. TabCAT brain health assessment
102. Task switching tests
103. Technology activities of daily living questionnaire (T-ADLQ)
104. Telephone interview for cognitive status (TICS)
105. Test of everyday attention (TEA)
106. Test of variables of attention (TOVA)
107. The 2 and 7 test
108. Thurstone word fluency test (TWFT)
109. Trail making test (TMT) A or/and B
110. VCAP battery
111. Verbal fluency
112. WAIS-III letter number sequencing
113. Wechsler abbreviated scale of intelligence

114. Wechsler adult intelligence scale (revised)
115. Wechsler memory scale
116. Wechsler test of adult reading
117. Western aphasia battery revised (WAB-AQ)
118. Wide range achievement test (WRAT)
119. Wisconsin Card Sort Test

Additional cognitive tests not previously mentioned[whitespace]



Lee A, *et al. BMJ Open* 2024; 14:e080334. doi: 10.1136/bmjopen-2023-080334

## Supplementary tables and figures

**Table 1:** Number of studies using different categories of brain health measurements*

| Type of study | Number of studies | Imaging | Cognitive | Mental health | Clinical | Bio | >1 category (% of number of studies) |
|---|---|---|---|---|---|---|---|
| **Case series** | 25 | 19 | 15 | 0 | 6 | 4 | 18 (72) |
| Cohort study | 609 | 471 | 318 | 11 | 132 | 73 | 382 (63) |
| Randomised controlled trial | 59 | 47 | 35 | 1 | 11 | 3 | 34 (58) |
| Narrative review | 11 | 7 | 7 | 0 | 3 | 6 | 10 (91) |
| Systematic review/meta-analysis | 19 | 15 | 12 | 1 | 8 | 7 | 16 (84) |
| Other | 4 | 2 | 3 | 0 | 1 | 1 | 3 (75) |

*Row data is not mutually exclusive as many studies used more than 1 category of methods

**Table 2:** Number of studies published by year using different categories of brain health measurements*

| Year | No. of studies published | Imaging | Cognitive | Mental health | Clinical | Biological | No. of multicategory studies |
|---|---|---|---|---|---|---|---|
| **2003** | 1 | 1 | 0 | 0 | 0 | 0 | 0 |
| **2007** | 1 | 1 | 1 | 0 | 0 | 0 | 1 |
| **2008** | 2 | 1 | 1 | 0 | 0 | 0 | 0 |
| **2009** | 5 | 4 | 4 | 0 | 2 | 1 | 3 |
| **2010** | 3 | 3 | 1 | 0 | 0 | 0 | 1 |
| **2011** | 6 | 5 | 4 | 0 | 4 | 2 | 2 |
| **2012** | 8 | 6 | 4 | 0 | 5 | 1 | 4 |
| **2013** | 16 | 14 | 10 | 0 | 2 | 5 | 6 |
| **2014** | 19 | 23 | 10 | 0 | 3 | 2 | 7 |
| **2015** | 34 | 33 | 16 | 0 | 4 | 5 | 16 |
| **2016** | 30 | 31 | 22 | 0 | 4 | 4 | 12 |
| **2017** | 54 | 53 | 32 | 2 | 6 | 6 | 22 |
| **2018** | 71 | 78 | 42 | 1 | 18 | 6 | 26 |
| **2019** | 83 | 84 | 63 | 2 | 22 | 6 | 41 |
| **2020** | 87 | 85 | 57 | 1 | 20 | 12 | 37 |
| **2021** | 115 | 115 | 62 | 2 | 30 | 10 | 44 |
| **2022** | 181 | 167 | 141 | 5 | 56 | 39 | 72 |
| **2023** | 11 | 8 | 4 | 0 | 3 | 3 | 11 |

*Row data is not mutually exclusive as many studies used more than 1 category of methods



Lee A, *et al. BMJ Open* 2024; 14:e080334. doi: 10.1136/bmjopen-2023-080334

**Table 3:** Number of brain health studies by country using different categories of brain health measurements. Row data is mutually exclusive.

| Country | Imaging | Cognitive | Mental health | Clinical | Bio-logical | Multi-category | Number of studies (%)* |
|---|---|---|---|---|---|---|---|
| **United States** | 93 | 24 | 0 | 8 | 6 | 221 | 354 (48.7) |
| **United Kingdom** | 19 | 2 | 0 | 3 | 0 | 43 | 67 (9.2) |
| **Canada** | 23 | 1 | 0 | 2 | 0 | 35 | 61 (8.4) |
| **Australia** | 7 | 3 | 0 | 2 | 0 | 17 | 29 (4.0) |
| **China#** | 6 | 2 | 0 | 2 | 0 | 12 | 22 (3.0) |
| **France** | 3 | 1 | 0 | 0 | 0 | 14 | 18 (2.5) |
| **Japan** | 5 | 2 | 0 | 1 | 0 | 7 | 15 (2.0) |
| **Netherlands** | 1 | 2 | 0 | 0 | 0 | 11 | 14 (1.9) |
| **South Korea** | 5 | 0 | 0 | 0 | 1 | 6 | 12 (1.7) |
| **Germany** | 2 | 0 | 0 | 0 | 0 | 10 | 12 (1.7) |
| **Norway** | 2 | 0 | 0 | 0 | 0 | 7 | 9 (1.2) |
| **Republic of Ireland** | 1 | 1 | 0 | 0 | 0 | 7 | 9 (1.2) |
| **Spain** | 2 | 2 | 0 | 1 | 1 | 2 | 8 (1.1) |
| **Brazil** | 1 | 0 | 0 | 0 | 0 | 6 | 7 (1.0) |
| **Sweden** | 1 | 0 | 0 | 0 | 0 | 6 | 7 (1.0) |
| **Taiwan** | 1 | 0 | 0 | 0 | 0 | 5 | 6 (0.8) |
| **Czech Republic** | 2 | 0 | 0 | 0 | 0 | 3 | 5 (0.7) |
| **Italy** | 0 | 0 | 0 | 0 | 0 | 5 | 5 (0.7) |
| **Iran#** | 1 | 1 | 0 | 0 | 0 | 2 | 4 (0.6) |
| **Hong Kong** | 1 | 0 | 0 | 0 | 0 | 2 | 3 (0.4) |
| **Portugal** | 1 | 0 | 0 | 0 | 0 | 2 | 3 (0.4) |
| **Switzerland** | 1 | 1 | 0 | 0 | 0 | 1 | 3 (0.4) |
| **Belgium** | 0 | 0 | 0 | 0 | 0 | 3 | 3 (0.4) |
| **Malaysia#** | 2 | 0 | 0 | 0 | 0 | 0 | 2 (0.3) |
| **Argentina#** | 1 | 0 | 0 | 0 | 0 | 1 | 2 (0.3) |
| **Israel** | 1 | 0 | 0 | 1 | 0 | 0 | 2 (0.3) |
| **Finland** | 0 | 0 | 0 | 1 | 0 | 1 | 2 (0.3) |
| **New Zealand** | 0 | 1 | 0 | 0 | 0 | 1 | 2 (0.3) |
| **Cuba#** | 1 | 0 | 0 | 0 | 0 | 0 | 1 (0.1) |
| **Pakistan#** | 1 | 0 | 0 | 0 | 0 | 0 | 1 (0.1) |
| **Thailand#** | 1 | 0 | 0 | 0 | 0 | 0 | 1 (0.1) |
| **India#** | 0 | 0 | 0 | 0 | 0 | 1 | 1 (0.1) |
| **South Africa#** | 0 | 1 | 0 | 0 | 0 | 0 | 1 (0.1) |
| **Tanzania#** | 0 | 0 | 0 | 0 | 0 | 1 | 1 (0.1) |
| **Denmark** | 1 | 0 | 0 | 0 | 0 | 0 | 1 (0.1) |
| **Greece** | 1 | 0 | 0 | 0 | 0 | 0 | 1 (0.1) |
| **Hungary** | 1 | 0 | 0 | 0 | 0 | 0 | 1 (0.1) |
| **Iceland** | 1 | 0 | 0 | 0 | 0 | 0 | 1 (0.1) |
| **Chile** | 0 | 0 | 0 | 0 | 0 | 1 | 1 (0.1) |

*Only non-review articles included in this table
# denotes lower or middle income country as defined by the World Bank



Lee A, *et al. BMJ Open* 2024; 14:e080334. doi: 10.1136/bmjopen-2023-080334

**Table 4:** Structural MRI methods for brain health measurement*

| Measurement method | Number of studies using this method (% of total studies) |
|---|---|
| *Structural MRI* | |
| Grey matter volume in specific region(s) | 133 (18.3) |
| White matter hyperintensities (WMHs) | 133 (18.3) |
| Total brain volume | 132 (18.2) |
| Whole brain grey matter volume | 106 (14.6) |
| Hippocampal volume | 105 (14.4) |
| White matter volume in specific region(s) | 95 (13.1) |
| Whole brain white matter volume | 77 (10.6) |
| Cortical thickness | 54 (7.4) |
| White matter lesion volume | 29 (4.0) |
| Cerebrospinal fluid volume in specific region(s) | 19 (2.6) |
| Cerebral microbleeds | 16 (2.2) |
| Lacunes | 4 (0.6) |
| Cortical superficial siderosis | 2 (0.3) |
| Lacunar infarcts | 2 (0.3) |
| Embolic infarcts | |
| Small vessel disease | 2 (0.3) |
| Perivascular spaces | 2 (0.3) |
| | |
| *Functional MRI* | |
| Cerebral blood flow in specific region(s) | 51 (7.0) |
| Resting state functional connectivity | 45 (6.2) |
| Task based functional connectivity | 31 (4.3) |
| Cerebral metabolic rate of oxygen | 2 (0.3) |
| | |
| *Diffusion MRI* | |
| Fractional anisotropy | 102 (14.0) |
| Mean diffusivity | 59 (8.1) |
| Axial diffusivity | 19 (2.6) |
| Radial diffusivity | 19 (2.6) |
| Free water | 4 (0.6) |
| Fibre bundle lengths | 3 (0.4) |
| Fibre density | 3 (0.4) |
| | |
| *Compound indices* | |
| Brain age gap calculations | 13 (1.8) |
| Brain atrophy and lesion index (BALI) | 8 (1.1) |
| Brain age estimations (MRI based) | 2 (0.3) |
| Brain health quotients (BHQ) | 2 (0.3) |
| Spatial pattern of atrophy for recognition of brain aging (SPARE-BA) | 2 (0.3) |
| | |
| *Miscellaneous imaging* | |
| PET amyloid load or presence | 38 (5.2) |
| PET tau | 11 (1.5) |
| Transcranial doppler ultrasound | 9 (1.2) |

| | |
|---|---|
| Transcranial magnetic stimulation (TMS) | 9 (1.2) |
| Functional near infrared spectroscopy (fNIRS) | 8 (1.1) |
| PET FDG | 7 (1.0) |
| Magnetoencephalography (MEG) – functional connectivity | 7 (1.0) |
| MRS N-acetylaspartate | 5 (0.7) |
| MRS glutamate | 6 (0.8) |
| MRS glutamine | 4 (0.6) |
| MRS lactate | 4 (0.6) |
| Duplex ultrasonography of carotid arteries | 4 (0.6) |
| PET florbetapir | 3 (0.4) |
| Magnetic resonance elastography (MRE) | 3 (0.4) |
| Pulsatility index | 3 (0.4) |
| Iron content (quantitative susceptibility weighted MRI) | 3 (0.4) |
| Myelin water fraction maps (MWF) | 2 (0.3) |

*Rows in the table are not mutually exclusive as many studies used more than one method of measurement.



Lee A, *et al. BMJ Open* 2024; 14:e080334. doi: 10.1136/bmjopen-2023-080334

**Table 5:** Cognitive tests for brain health measurement

| Measurement method | Number of studies using this method (% of total studies) |
|---|---|
| Trail making test (TMT) A or/and B | 86 (11.8) |
| Mini-mental status examination (MMSE) | 73 (10.0) |
| Stroop test | 64 (8.8) |
| Rey Auditory verbal learning test (RAVLT) (inc. modified) | 50 (6.9) |
| Montreal cognitive assessment (MoCA) | 48 (6.6) |
| Digit span | 39 (5.4) |
| Digit symbol substitution test (DSST) | 36 (5.0) |
| Verbal fluency | 32 (4.4) |
| Wechsler adult intelligence scale (inc. modified) | 32 (4.4) |
| Hopkins verbal learning test (HVLT) | 24 (3.3) |
| Wechsler memory scale | 22 (3.0) |
| California verbal learning test (CVLT-II) | 20 (2.8) |
| Logical memory | 19 (2.6) |
| Boston naming test (BNT) | 17 (2.3) |
| Rey-Osterrieth Complex Figure test (CFT) | 16 (2.2) |
| Symbol digit modalities test (SDMT) | 15 (2.1) |
| TabCAT UCSF brain health assessment | 15 (2.1) |
| Wechsler abbreviated scale of intelligence | 15 (2.1) |
| Cogstate brief battery (CBB) | 13 (1.8) |
| Controlled oral word association test | 12 (1.7) |
| Delis-Kaplan executive functioning (D-KEFS) | 12 (1.7) |
| Animal naming | 11 (1.5) |
| Repeatable battery for the assessment of neuropsychological status (RBANS) | 11 (1.5) |
| Clinical dementia rating (CDR) | 10 (1.4) |
| CERAD word list delayed recall and memory battery | 9 (1.2) |
| National adult reading test | 9 (1.2) |
| Reaction time tasks | 9 (1.2) |
| Everyday cognition scale (ECog) | 8 (1.1) |
| Task switching tests | 8 (1.1) |
| Benton Visual Retention Test (BVRT) | 7 (1.0) |
| Grooved pegboard (PEGS) | 7 (1.0) |
| N- back working memory task | 7 (1.0) |
| National institutes of health (NIH) Toolbox-cognitive battery | 7 (1.0) |
| Category fluency | 6 (0.8) |
| Clock drawing test (CDT) | 6 (0.8) |
| Spatial working memory | 6 (0.8) |
| Wechsler test of adult reading | 6 (0.8) |
| CANTAB intra extra dimensional set shift (IED) | 5 (0.7) |
| CNS vital signs | 5 (0.7) |
| Color trails test | 5 (0.7) |
| Flanker NIH Toolbox | 5 (0.7) |
| Hooper visual organisation test | 5 (0.7) |
| Letter fluency | 5 (0.7) |
| Letter number sequencing | 5 (0.7) |
| Selective reminding test | 5 (0.7) |
| Visual memory | 5 (0.7) |
| Behaviour rating inventory of executive function adult version (BRIEF-A) | 4 (0.6) |
| Brief visuospatial memory test (BVMT) (inc.modified) | 4 (0.6) |
| DKEFS Color Word Interference | 4 (0.6) |



Lee A, *et al. BMJ Open* 2024; 14:e080334. doi: 10.1136/bmjopen-2023-080334

| | |
|---|---|
| Global cognitive function (GCF) score | 4 (0.6) |
| Mattis dementia rating scale (DRS) | 4 (0.6) |
| Alzheimer's disease assessment scale (ADAS-cog) | 4 (0.6) |
| Paired associates' task | 4 (0.6) |
| Pattern comparison processing speed (PCPS) | 4 (0.6) |
| Visual reproduction | 4 (0.6) |
| Digit symbol coding | 4 (0.6) |
| Brain health test (BHT) | 4 (0.6) |
| Cogniciti's brain health assessment (BHA) | 3 (0.4) |
| Bell cancellation test | 3 (0.4) |
| Brief cognitive ability measure (B-CAM) | 3 (0.4) |
| Cogstate neurocognitive battery | 3 (0.4) |
| Edinburgh Handedness Inventory (EHI) | 3 (0.4) |
| Eriksen-Flanker task | 3 (0.4) |
| Face name association test | 3 (0.4) |
| Memtrax memory test | 3 (0.4) |
| MiniCog | 3 (0.4) |
| Mnemonic similarity task (MST) | 3 (0.4) |
| Preclinical Alzheimer's cognitive composite 5 (PACC5) | 3 (0.4) |
| Telephone interview for cognitive status (TICS) | 3 (0.4) |
| Test of variables of attention (TOVA) | 3 (0.4) |
| Thurstone word fluency test (TWFT) | 3 (0.4) |
| VCAP battery | 3 (0.4) |
| Wide range achievement test (WRAT) | 3 (0.4) |
| Wisconsin Card Sort Test | 3 (0.4) |
| Dimensional change card sort (DCCS) | 3 (0.4) |
| Four Choice reaction time | 3 (0.4) |
| Go/No go | 3 (0.4) |
| Oral reading recognition (ORR) | 3 (0.4) |
| Pairs matching | 3 (0.4) |
| Picture Sequence Memory (PSM) | 3 (0.4) |
| Semantic fluency | 3 (0.4) |
| Spatial reconstruction task | 3 (0.4) |
| Study specific neuropsychological test battery (unnamed) | 3 (0.4) |
| Working memory | 3 (0.4) |
| Attention network test (ANT) | 2 (0.3) |
| Auditory consonant trigrams | 2 (0.3) |
| Cambridge Cognition Paired Associates Learning | 2 (0.3) |
| Color word interference test (CWIT) | 2 (0.3) |
| Continuous paired associative learning (CPAL) task | 2 (0.3) |
| Corsi block test | 2 (0.3) |
| Gerontology functional assessment tool (NCGG-FAT) | 2 (0.3) |
| Hasegawa's dementia scale-revised (HDS-R) | 2 (0.3) |
| Lifetime of experiences questionnaire (LEQ) | 2 (0.3) |
| Mayo clinic study of aging (MCSA) | 2 (0.3) |
| Positive and negative affect schedule (PANAS) | 2 (0.3) |
| Sustained attention to response task (SART) | 2 (0.3) |
| Western aphasia battery revised (WAB-AQ) | 2 (0.3) |
| 7-minute screening test | 2 (0.3) |
| Addenbrooke's Cognitive Examination (ACE-III) | 2 (0.3) |
| Benson Figure | 2 (0.3) |
| Cambridge Neurological Test Automated Battery (CANTAB) | 2 (0.3) |
| Card rotations test | 2 (0.3) |
| Cognitive Activity questionnaire | 2 (0.3) |
| Digit vigilance | 2 (0.3) |
| Fluid intelligence | 2 (0.3) |
| Forward and reverse memory span | 2 (0.3) |



Lee A, *et al. BMJ Open* 2024; 14:e080334. doi: 10.1136/bmjopen-2023-080334

| | |
|---|---|
| Identical pictures test | 2 (0.3) |
| Lifetime Experience Questionnaire | 2 (0.3) |
| List sorting working memory (LSWM) | 2 (0.3) |
| NCGG-FAT (National Center for Geriatrics and Gerontology Functional Assessment Tool) | 2 (0.3) |
| Neuro-QoL | 2 (0.3) |
| Paper folding task | 2 (0.3) |
| Picture Vocabulary Test (PVT) | 2 (0.3) |
| Preclinical Alzheimer's Cognitive score | 2 (0.3) |
| Prospective memory | 2 (0.3) |
| Story recall | 2 (0.3) |
| Visual Inspection Time | 2 (0.3) |
| Tower of London | 2 (0.3) |

*Rows in the table are not mutually exclusive as many studies used more than one method of measurement.



Lee A, *et al. BMJ Open* 2024; 14:e080334. doi: 10.1136/bmjopen-2023-080334

**Table 6:** Biological methods for brain health measurement

| Measurement method | Number of studies using this method (% of total studies) |
|---|---|
| Blood ApoE4 genotype | 41 (5.6) |
| Blood brain-derived neurotrophic factor (BDNF) | 33 (4.5) |
| CSF neurofilament light | 11 (1.5) |
| CSF tau levels | 10 (1.4) |
| Blood Aß 42 or 40 | 9 (1.2) |
| Blood tau levels | 7 (1.0) |
| Post-mortem neuropathological evaluation of brain tissue | 4 (0.6) |
| Blood S100Beta levels | 3 (0.4) |
| Blood vascular endothelial growth factor (VEGF) | 3 (0.4) |
| CSF BDNF levels | 2 (0.3) |
| Blood interleukin-8 (IL8) levels | 2 (0.3) |
| Blood glial fibrillary acidic protein (GFAP) levels | 2 (0.3) |
| Blood tumour necrosis factor alpha levels | 2 (0.3) |
| Blood eotaxin levels | 2 (0.3) |
| Blood macrophage inflammatory protein-1alpha levels | 2 (0.3) |

*Rows in the table are not mutually exclusive as many studies used more than one method of measurement.

**Table 7:** Clinical methods for brain health measurement

| **Measurement method** | **Number of studies using this method (% of total studies)** |
|---|---|
| Electroencephalography (EEG) | 24 (3.3) |
| Lifestyle for brain health (LIBRA) index | 12 (1.7) |
| EEG based brain age | 5 (0.7) |
| Mindreader (EEG software) | 4 (0.6) |
| Clinical diagnosis of Alzheimer's disease or dementia | 4 (0.6) |
| Hand grip strength | 4 (0.6) |
| Pittsburgh Sleep Quality Index (PSQI) | 3 (0.4) |
| RAND-36 health related quality of life survey | 2 (0.3) |
| SF-36 (short form survey) | 2 (0.3) |
| Test of premorbid functioning | 2 (0.3) |

*Rows in the table are not mutually exclusive as many studies used more than one method of measurement.



Lee A, *et al. BMJ Open* 2024; 14:e080334. doi: 10.1136/bmjopen-2023-080334

**Table 8:** Mental health methods for brain health measurement

| **Measurement method** | **Number of studies using this method (% of total studies)** |
|---|---|
| Barratt impulsiveness scale | 11 (1.5) |
| Geriatric depression scale | 7 (1.0) |
| Patient health questionnaire (PHQ)-9 depression scale | 5 (0.7) |
| Hospital anxiety and depression (HADS) scales | 5 (0.7) |
| Beck depression inventory | 5 (0.7) |
| Centre for epidemiologic studies depression scale (CES-D) | 4 (0.6) |
| Perceived stress scale | 3 (0.4) |
| PHQ-8 depression scale | 3 (0.4) |
| Generalised anxiety disorder assessment (GAD)-7 | 3 (0.4) |
| Ruminative responses scale | 2 (0.3) |
| Post-traumatic stress disorder checklist for DSM-V[#] (PCL-5) | 2 (0.3) |

*Rows in the table are not mutually exclusive as many studies used more than one method of measurement. #Diagnostic and Statistical Manual of Mental Disorders, Fifth Edition



Lee A, *et al. BMJ Open* 2024; 14:e080334. doi: 10.1136/bmjopen-2023-080334
