# Supplementary figures and images for: Brain health measurement: a scoping review
Source: BMJ Open. 2024 Feb 10;14(2):e080334. doi: 10.1136/bmjopen-2023-080334 (PMC10862273; doi:10.1136/bmjopen-2023-080334)

## Number of brain health publications per year

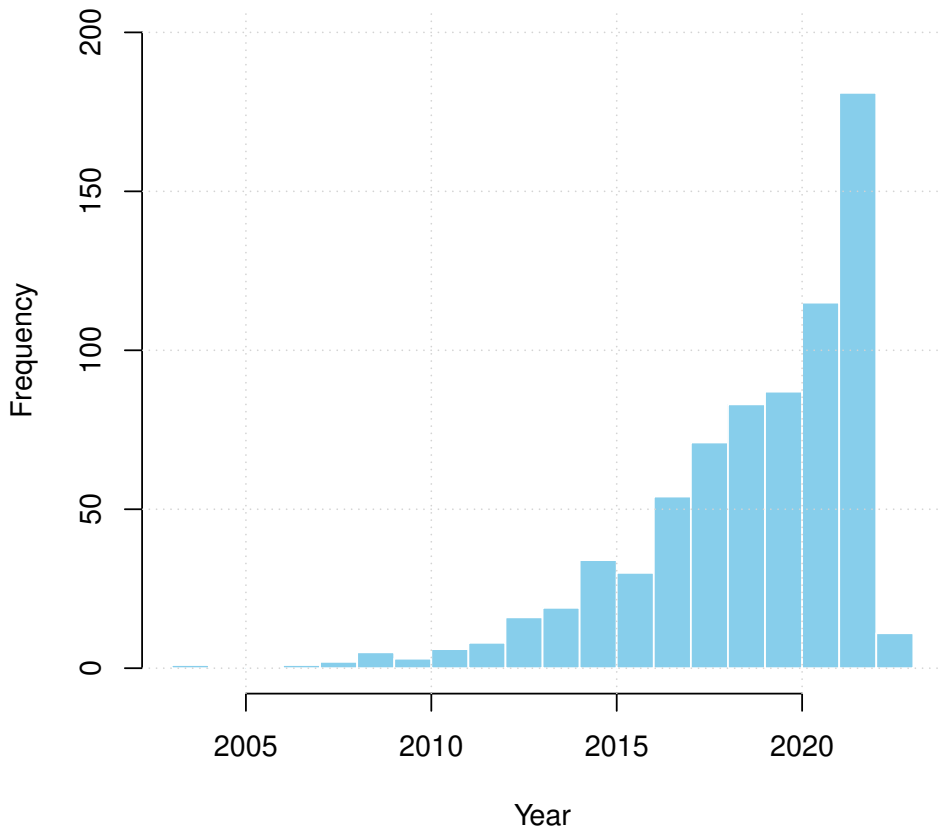

Supplement: Supplementary data [file bmjopen-2023-080334supp002.pdf]

Number of brain health studies by country

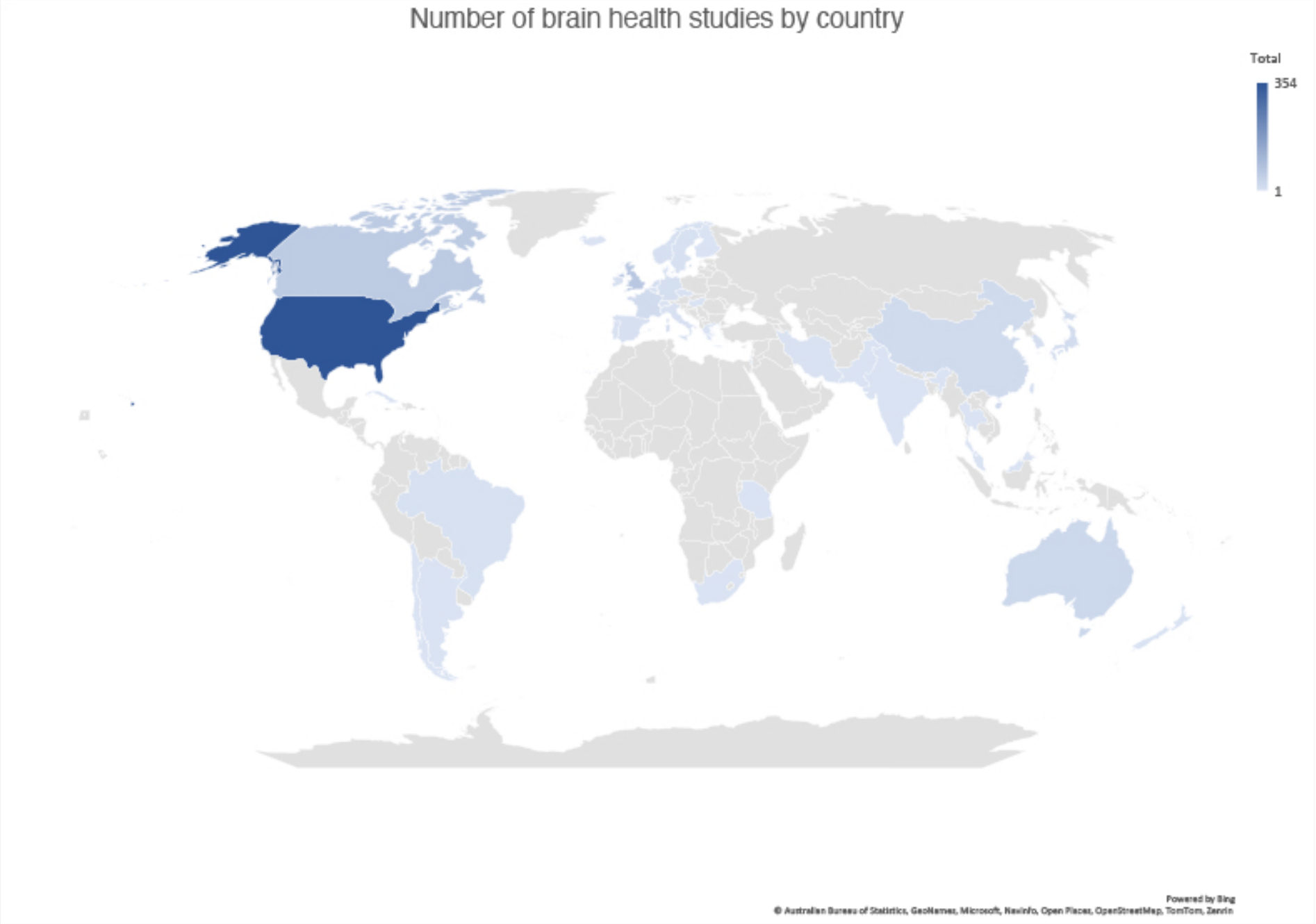

Supplement: Supplementary data [file bmjopen-2023-080334supp003.pdf]
