# Supplementary material for: Brain health measurement: a scoping review
Source: BMJ Open. 2024 Feb 10;14(2):e080334. doi: 10.1136/bmjopen-2023-080334 (PMC10862273; doi:10.1136/bmjopen-2023-080334)
Supplement: Author's manuscript [file bmjopen-2023-080334.draft_revisions.pdf]

BMJ Open is committed to open peer review. As part of this commitment we make the peer review history of every article we publish publicly available.

When an article is published we post the peer reviewers' comments and the authors' responses online. We also post the versions of the paper that were used during peer review. These are the versions that the peer review comments apply to.

The versions of the paper that follow are the versions that were submitted during the peer review process. They are not the versions of record or the final published versions. They should not be cited or distributed as the published version of this manuscript.

BMJ Open is an open access journal and the full, final, typeset and author-corrected version of record of the manuscript is available on our site with no access controls, subscription charges or pay-per-view fees (http://bmjopen.bmj.com).

If you have any questions on BMJ Open's open peer review process please email info.bmjopen@bmj.com

BMJ Open

Brain health measurement – a scoping review

Journal:	*BMJ Open*
Manuscript ID	bmjopen-2023-080334
Article Type:	Original research
Date Submitted by the Author:	03-Oct-2023
Complete List of Authors:	Lee, Angeline; University of Oxford, Nuffield Department of Population Health<br>Shah, Suraj; Health Education England London<br>Atha, Karyna; University of Oxford<br>Indoe, Peter; Health Education Thames Valley - HETV<br>Mahmoud, Naira; Health Education Thames Valley - HETV<br>Niblett, Guy; Health Education Thames Valley - HETV<br>Pradhan, Vidushi; Health Education Thames Valley - HETV<br>Roberts, Nia; University of Oxford, Bodleian Health Care Libraries,<br>Malouf, Reem; National Perinatal Epidemiology Unit, uffield Department of Population Health<br>Topiwala, Anya; University of Oxford, Nuffield Department of Population Health
Keywords:	Dementia, Aging, NEUROLOGY, Old age psychiatry < PSYCHIATRY, PUBLIC HEALTH, Aged, 80 and over

SCHOLARONE™
Manuscripts

I, the Submitting Author has the right to grant and does grant on behalf of all authors of the Work (as defined in the below author licence), an exclusive licence and/or a non-exclusive licence for contributions from authors who are: i) UK Crown employees; ii) where BMJ has agreed a CC-BY licence shall apply, and/or iii) in accordance with the terms applicable for US Federal Government officers or employees acting as part of their official duties; on a worldwide, perpetual, irrevocable, royalty-free basis to BMJ Publishing Group Ltd ("BMJ") its licensees and where the relevant Journal is co-owned by BMJ to the co-owners of the Journal, to publish the Work in this journal and any other BMJ products and to exploit all rights, as set out in our licence.

The Submitting Author accepts and understands that any supply made under these terms is made by BMJ to the Submitting Author unless you are acting as an employee on behalf of your employer or a postgraduate student of an affiliated institution which is paying any applicable article publishing charge ("APC") for Open Access articles. Where the Submitting Author wishes to make the Work available on an Open Access basis (and intends to pay the relevant APC), the terms of reuse of such Open Access shall be governed by a Creative Commons licence – details of these licences and which Creative Commons licence will apply to this Work are set out in our licence referred to above.

Other than as permitted in any relevant BMJ Author's Self Archiving Policies, I confirm this Work has not been accepted for publication elsewhere, is not being considered for publication elsewhere and does not duplicate material already published. I confirm all authors consent to publication of this Work and authorise the granting of this licence.

For peer review only

Brain health measurement – a scoping review

AUTHORS

Lee AH[1], Shah S[2], Atha K[3], Indoe P[4], Mahmoud N[4], Niblett G[4], Pradhan V[4], Roberts N[5], Malouf R[1], Topiwala A[1]

1. Big Data Institute, Nuffield Department of Population Health, University of Oxford
2. South London School of Anaesthesia, Health Education England
3. Oxford Medical School, University of Oxford
4. Health Education Thames Valley
5. Outreach Librarian Knowledge Centre, Bodleian Health Care Libraries, Oxford, UK.

ABSTRACT

Objective: To systematically evaluate brain health measurement in the scientific literature to date, informing development of a core outcome set.

Design: Scoping review

Introduction: Preservation of brain health is an urgent priority for the world's ageing population. The evidence base for brain health optimisation strategies is rapidly expanding, but clear recommendations have been limited by heterogeneity in measurement of brain health outcomes.

Methods: A broad and sensitive search strategy developed from the preliminary search was conducted on the Medline, APA PsycArticles and Embase databases was performed in January 2023. Studies were included if they described brain health evaluation methods in sufficient detail in human adults and were in English language. Two reviewers independently screened titles, abstracts and full texts for inclusion and extracted data using Covidence software.

Results: From 6987 articles identified by the search, 727 studies met inclusion criteria. Study publication increased by 22 times in the last decade. Cohort study was the most common study design (n=609,84%). 479 unique methods of measuring brain health were identified, comprising imaging, cognitive, mental health, biological and clinical categories. Seven of the top ten most frequently used brain health measurement methods were imaging-based, including structural imaging of grey matter and hippocampal volumes and white matter hyperintensities. Cognitive tests such as the trail making test accounted for 286 (59.7%) of all brain health measurement methods.

Conclusions: The scientific literature surrounding brain health has increased exponentially, yet measurement methods are highly heterogeneous across studies which may explain lack clinical translation. Future studies should aim to progress core outcome set development and broaden from the current focus on neuroimaging outcomes to include a range of outcomes.

STRENGTHS AND LIMITATIONS OF THIS STUDY

- Broad search strategy developed after a preliminary search of the current evidence base
- Wide inclusion criteria to capture maximal number of relevant studies
- Protocol does not include description of risk of bias for included studies
- Non-English language articles were excluded

For peer review only

INTRODUCTION

Brain health can be defined as the preservation of optimal brain integrity and mental and cognitive function at a given age in the absence of overt brain diseases that affect normal brain function.(1) The ageing population in the world is increasing and the number of people aged over 60 is expected to grow to 2 billion in 2050 (2).

The Global Burden of Disease study 2013 demonstrated that neurological disorders are a leading cause of chronic disorders worldwide, and that the years lived with disability for all neurological disorders increased by 59.6% from 1990 to 2013 as people are living for longer. The years lived with disability for Alzheimer's disease alone increased by 91.8% from 1990 to 2013 (3). Ten years on, the burden of disease has increased even further. In May 2022, the World Health Organisation member states implemented a global action plan to improve healthcare and wellbeing of people living with neurological disorders and reducing mortality, morbidity and disability associated with these conditions (4).

The time is ripe to invest in methods of improving and optimising brain health to maximise the population quality of life and minimise disability, disease and death related to neurological diseases (1).

The research world has responded by launching many studies to trial interventions to preserve brain health, but the wide variation in the methods used to study brain health is limiting comparison between studies (5) and therefore recommendations for interventions that can potentially improve brain health (6). This has led to wasteful research practices – including repetition of studies comparing similar interventions but measuring different outcomes (5, 7). There is no consensus on a set of brain health outcomes that would be meaningful and important to patients, nor is there one on how specific outcomes should be measured and reported. There is an urgent need to achieve a consensus in brain health reporting to encourage prevention, optimisation and potentially even treatment for neurological diseases.

We aimed to conduct a systematic scoping review to evaluate methods of brain health measurement in current literature. Core outcome sets (COS) are agreed standardised sets of outcomes that should be measured and reported, as a minimum, in all clinical trials in specific areas of health or healthcare(5). These could be extended to include other types of study design. Despite the introduction of organisations such as the Core Outcome Measures in Effectiveness Trials (COMET) initiative in 2010 and support from various organisations to boost COS use in research, COS uptake is low in many branches of research including brain health(8). A scoping review was chosen as the best technique to perform an initial rapid mapping of current evidence on brain health and identify the most used brain health outcome measures, to inform future consensus work on brain health outcomes to facilitate development of a brain health COS.

METHODS

This scoping review was conducted in accordance with the Preferred Reporting Items for Systematic reviews and meta-analyses extension for scoping reviews (PRISMA-ScR) checklist (9).

A preliminary search of Medline, the Cochrane Database of Systematic Reviews and JBI Evidence Synthesis was conducted, and no current or underway systematic reviews or scoping reviews were identified on this topic. Over 3000 papers were found on the preliminary Medline search with the

For peer review only - http://bmjopen.bmj.com/site/about/guidelines.xhtml

search terms ("brain-health" OR “cognitive-health”) AND ("measur*" OR "outcome*" OR "biomarker" OR "marker"), so there was sufficient evidence available to inform this review.

Inclusion/exclusion criteria

Studies that met the following criteria were included in the review:

1. Participants must be human
2. Participants must be aged 18 years or over
3. Studies must report outcomes that are measuring ‘brain health’
4. Studies must be written in the English language

Studies were excluded from the review if they did not report brain health measures with sufficient detail to enable replication, for example studies that reported that imaging was used without specifying fractional anisotropy as the measurement.

The human brain develops significantly between childhood and adulthood, with different structure, network organisation and function (10, 11). Studies about children or adolescents were excluded as brain health measurement tools in children may not be suitable for adults and vice versa. Brain health is a human concept due to the complexity of human brain functions; therefore, we excluded studies on non-humans.

Search strategy

A systematic search was conducted of Medline, Embase and APA PsycArticles databases for articles published from the inception of each of these databases to present using a search strategy developed with an information specialist (Appendix I). The syntax of the search strategy was modified for use with Embase and APA PsycArticles.

Due to the relatively new concept of brain health, the search strategy was informed by an initial limited search of Medline. The following search terms were used: ("brain-health" OR “cognitive-health”) AND ("measur*" OR "outcome*" OR "biomarker" OR "marker") on 12th December 2022, and 2362 results were screened by one author, of which 72 full text papers were found to be suitable for inclusion for the review. The Yale MeSH analyser was used to extract all MeSH headings and author keywords used in these 72 full text papers. The terms were analysed with Rstudio (Version: 2022.12.0+353 (2022.12.0+353)). 1035 search terms were used in the 72 papers, with 286 distinct search terms. All terms were considered for inclusion into the search strategy (Appendix I).

This scoping review considered all study designs, both experimental and quasi-experimental study designs including randomized controlled trials, non-randomized controlled trials, before and after studies and interrupted time-series studies. In addition, analytical observational studies including prospective and retrospective cohort studies, case-control studies and analytical cross-sectional studies were considered for inclusion. This review also considered descriptive observational study designs including case series, individual case reports and descriptive cross-sectional studies for inclusion. Systematic reviews, text and opinion papers and conference abstracts that met the inclusion criteria were considered, depending on the research question.

Source of evidence selection

For peer review only - http://bmjopen.bmj.com/site/about/guidelines.xhtml

Following the search on 25th January 2023, all identified citations were uploaded into Covidence (Covidence systematic review software, Veritas Health Innovation, Melbourne, Australia. Available at www.covidence.org.), which is a web-based collaboration software platform that streamlines the production of systematic and other literature reviews. Duplicates were removed by Covidence during this process, and further duplicates were manually removed.

Following a pilot test, two independent reviewers screened each title and abstract for assessment against the inclusion criteria for the review. Full-text articles for potentially relevant sources were imported into Covidence, and these were assessed in detail against the inclusion criteria by two independent reviewers. Reasons for exclusion of sources of evidence at full text that did not meet the inclusion criteria were recorded by the system. Conflicts in reviewer opinion were all resolved through discussion, although an additional independent reviewer (AT) was available for adjudication.

The results of the search and the study inclusion process are presented in a Preferred Reporting Items for Systematic Reviews and Meta-analyses extension for scoping review (PRISMA-ScR) flow diagram (9) (Appendix II).

Data extraction

Data was extracted from included papers by two independent reviewers using a data extraction template developed by the reviewers on Covidence (Appendix III). All conflicts were resolved through discussion before the data extraction process was finalised. All brain health measurement methods were grouped into categories and tabulated based on frequency of use.

RESULTS

A total of 6155 studies were included in the title and abstract screening after removing duplicates from the original search results. After abstract review, 924 studies were assessed for eligibility using the inclusion and exclusion criteria, leaving 727 studies for data extraction (Appendix II).

There were 609 (83.8%) cohort studies, 59 (8.1%) randomised controlled trials or sub studies within randomised controlled trials; 25 (3.4%) case series; 19 (2.6%) systematic reviews with or without meta-analyses; 11 (1.5%) narrative reviews and 4 (0.6%) were other study types.

The range of years of publication of brain health studies was between year 2003 and 2023. Supplemental figure 1 shows a histogram of number of brain health publications per year. The number of published brain health studies is steadily increasing and has more than tripled in the last 5 years (54 papers published in 2017 and 181 papers published in 2022), and 22 times in the last 10 years (8 papers published in 2012).

Brain health measurement methods

There were 478 unique methods of brain health measurement identified in the data extraction. Two hundred and sixty-eight (56.1%) of these were only used once, and these are presented in table form in Supplementary Appendix 1. The remaining 210 methods will be presented in imaging, biological, clinical, mental health, and cognitive test categories.

Within these categories, 1 study (0.1%) included outcome measures from 4 categories (cognitive, mental health, clinical and biological); 34 studies (4.7%) included measures from 3 categories (most commonly imaging, cognitive and biological); 233 studies (32.0%) included measures from 2

For peer review only - http://bmjopen.bmj.com/site/about/guidelines.xhtml

categories (most commonly imaging and cognitive); and the remaining 460 studies (63.3%) included measures from one of these categories (most commonly imaging).

Eight of the top ten most prevalent methods for measuring brain health were imaging based. These were mainly volume estimates for gray and white matter in specific regions, particularly the hippocampus, and the whole brain; presence of white matter hyperintensities, and fractional anisotropy. The trail making test and mini-mental status examination (MMSE) were the other two most prevalent methods.

Imaging

Imaging was the most common method of brain health measurement (514 studies, 70.7%), particularly magnetic resonance imaging (MRI) based measures. Within imaging, measurements were divided into structural, functional, diffusion MRI parameters, compound imaging indices, and miscellaneous forms of imaging (Table 1).

Approximately a fifth of all studies in our review utilised structural MRI based volumetric estimates, particularly of grey matter and hippocampal volumes; or looked for the presence of white matter hyperintensities. Seven percent of studies looked at cerebral blood flow in specific regions of the brain using functional MRI techniques at rest or whilst performing tasks. Brain age gap calculations comparing an imaging estimate of brain age derived from various MRI parameters to a person's chronological age were used in 1.8% of studies. Positron emission tomography (PET) measured amyloid load or presence was the most used (5.2%) type of non-MRI imaging method to measure brain health.

Table 1: Structural MRI methods for brain health measurement

Measurement method	Number of studies using this method (%)
Structural MRI	
Grey matter volume in specific region(s)	133 (18.3)
White matter hyperintensities (WMHs)	133 (18.3)
Total brain volume	132 (18.2)
Whole brain grey matter volume	106 (14.6)
Hippocampal volume	105 (14.4)
White matter volume in specific region(s)	95 (13.1)
Whole brain white matter volume	77 (10.6)
Cortical thickness	54 (7.4)
White matter lesion volume	29 (4.0)
Cerebrospinal fluid volume in specific region(s)	19 (2.6)
Cerebral microbleeds	16 (2.2)
Lacunes	4 (0.6)
Cortical superficial siderosis	2 (0.3)
Lacunar infarcts	2 (0.3)
Embolic infarcts	
Small vessel disease	2 (0.3)
Perivascular spaces	2 (0.3)
Functional MRI	

For peer review only - http://bmjopen.bmj.com/site/about/guidelines.xhtml

Cerebral blood flow in specific region(s)	51 (7.0)
Resting state functional connectivity	45 (6.2)
Task based functional connectivity	31 (4.3)
Cerebral metabolic rate of oxygen	2 (0.3)
Diffusion MRI	
Fractional anisotropy	102 (14.0)
Mean diffusivity	59 (8.1)
Axial diffusivity	19 (2.6)
Radial diffusivity	19 (2.6)
Free water	4 (0.6)
Fibre bundle lengths	3 (0.4)
Fibre density	3 (0.4)
Compound indices	
Brain age gap calculations	13 (1.8)
Brain atrophy and lesion index (BALI)	8 (1.1)
Brain age estimations (MRI based)	2 (0.3)
Brain health quotients (BHQ)	2 (0.3)
Spatial pattern of atrophy for recognition of brain aging (SPARE-BA)	2 (0.3)
Miscellaneous imaging	
PET amyloid load or presence	38 (5.2)
PET tau	11 (1.5)
Transcranial doppler ultrasound	9 (1.2)
Transcranial magnetic stimulation (TMS)	9 (1.2)
Functional near infrared spectroscopy (fNIRS)	8 (1.1)
PET FDG	7 (1.0)
Magnetoencephalography (MEG) – functional connectivity	7 (1.0)
MRS N-acetylaspartate	5 (0.7)
MRS glutamate	6 (0.8)
MRS glutamine	4 (0.6)
MRS lactate	4 (0.6)
Duplex ultrasonography of carotid arteries	4 (0.6)
PET florbetapir	3 (0.4)
Magnetic resonance elastography (MRE)	3 (0.4)
Pulsatility index	3 (0.4)
Iron content (quantitative susceptibility weighted MRI)	3 (0.4)
Myelin water fraction maps (MWF)	2 (0.3)

Cognitive tests

Three-hundred and thirty (45.4%) studies used a form of cognitive test when measuring brain health. The highest number of individual brain health measurement methods used more than once were in this category (115/210, 54.8%). Only named test batteries or tests described in sufficient detail for replication were included in the data extraction.

The trail making test A or B, mini-mental status examination and Stroop tests were the most used of all cognitive tests, with approximately a tenth of all studies using one or more of these in evaluating brain health (Table 2).

For peer review only - http://bmjopen.bmj.com/site/about/guidelines.xhtml

Table 2: Cognitive tests for brain health measurement

Measurement method	Number of studies using this method (%)
Trail making test (TMT) A or/and B	86 (11.8)
Mini-mental status examination (MMSE)	73 (10.0)
Stroop test	64 (8.8)
Rey Auditory verbal learning test (RAVLT) (inc. modified)	50 (6.9)
Montreal cognitive assessment (MoCA)	48 (6.6)
Digit span	39 (5.4)
Digit symbol substitution test (DSST)	36 (5.0)
Verbal fluency	32 (4.4)
Wechsler adult intelligence scale (inc. modified)	32 (4.4)
Hopkins verbal learning test (HVLT)	24 (3.3)
Wechsler memory scale	22 (3.0)
California verbal learning test (CVLT-II)	20 (2.8)
Logical memory	19 (2.6)
Boston naming test (BNT)	17 (2.3)
Rey-Osterrieth Complex Figure test (CFT)	16 (2.2)
Symbol digit modalities test (SDMT)	15 (2.1)
TabCAT UCSF brain health assessment	15 (2.1)
Wechsler abbreviated scale of intelligence	15 (2.1)
Cogstate brief battery (CBB)	13 (1.8)
Controlled oral word association test	12 (1.7)
Delis-Kaplan executive functioning (D-KEFS)	12 (1.7)
Animal naming	11 (1.5)
Repeatable battery for the assessment of neuropsychological status (RBANS)	11 (1.5)
Clinical dementia rating (CDR)	10 (1.4)
CERAD word list delayed recall and memory battery	9 (1.2)
National adult reading test	9 (1.2)
Reaction time tasks	9 (1.2)
Everyday cognition scale (ECog)	8 (1.1)
Task switching tests	8 (1.1)
Benton Visual Retention Test (BVRT)	7 (1.0)
Grooved pegboard (PEGS)	7 (1.0)
N- back working memory task	7 (1.0)
National institutes of health (NIH) Toolbox-cognitive battery	7 (1.0)
Category fluency	6 (0.8)
Clock drawing test (CDT)	6 (0.8)
Spatial working memory	6 (0.8)
Wechsler test of adult reading	6 (0.8)
CANTAB intra extra dimensional set shift (IED)	5 (0.7)
CNS vital signs	5 (0.7)
Color trails test	5 (0.7)
Flanker NIH Toolbox	5 (0.7)
Hooper visual organisation test	5 (0.7)
Letter fluency	5 (0.7)
Letter number sequencing	5 (0.7)
Selective reminding test	5 (0.7)
Visual memory	5 (0.7)
Behaviour rating inventory of executive function adult version (BRIEF-A)	4 (0.6)
Brief visuospatial memory test (BVMT) (inc.modified)	4 (0.6)

For peer review only - http://bmjopen.bmj.com/site/about/guidelines.xhtml

DKEFS Color Word Interference	4 (0.6)
Global cognitive function (GCF) score	4 (0.6)
Mattis dementia rating scale (DRS)	4 (0.6)
Alzheimer’s disease assessment scale (ADAS-cog)	4 (0.6)
Paired associates’ task	4 (0.6)
Pattern comparison processing speed (PCPS)	4 (0.6)
Visual reproduction	4 (0.6)
Digit symbol coding	4 (0.6)
Brain health test (BHT)	4 (0.6)
Cogniciti's brain health assessment (BHA)	3 (0.4)
Bell cancellation test	3 (0.4)
Brief cognitive ability measure (B-CAM)	3 (0.4)
Cogstate neurocognitive battery	3 (0.4)
Edinburgh Handedness Inventory (EHI)	3 (0.4)
Eriksen-Flanker task	3 (0.4)
Face name association test	3 (0.4)
Memtrax memory test	3 (0.4)
MiniCog	3 (0.4)
Mnemonic similarity task (MST)	3 (0.4)
Preclinical Alzheimer's cognitive composite 5 (PACC5)	3 (0.4)
Telephone interview for cognitive status (TICS)	3 (0.4)
Test of variables of attention (TOVA)	3 (0.4)
Thurstone word fluency test (TWFT)	3 (0.4)
VCAP battery	3 (0.4)
Wide range achievement test (WRAT)	3 (0.4)
Wisconsin Card Sort Test	3 (0.4)
Dimensional change card sort (DCCS)	3 (0.4)
Four Choice reaction time	3 (0.4)
Go/No go	3 (0.4)
Oral reading recognition (ORR)	3 (0.4)
Pairs matching	3 (0.4)
Picture Sequence Memory (PSM)	3 (0.4)
Semantic fluency	3 (0.4)
Spatial reconstruction task	3 (0.4)
Study specific neuropsychological test battery (unnamed)	3 (0.4)
Working memory	3 (0.4)
Attention network test (ANT)	2 (0.3)
Auditory consonant trigrams	2 (0.3)
Cambridge Cognition Paired Associates Learning	2 (0.3)
Color word interference test (CWIT)	2 (0.3)
Continuous paired associative learning (CPAL) task	2 (0.3)
Corsi block test	2 (0.3)
Gerontology functional assessment tool (NCGG-FAT)	2 (0.3)
Hasegawa's dementia scale-revised (HDS-R)	2 (0.3)
Lifetime of experiences questionnaire (LEQ)	2 (0.3)
Mayo clinic study of aging (MCSA)	2 (0.3)
Positive and negative affect schedule (PANAS)	2 (0.3)
Sustained attention to response task (SART)	2 (0.3)
Western aphasia battery revised (WAB-AQ)	2 (0.3)
7-minute screening test	2 (0.3)
Addenbrooke's Cognitive Examination (ACE-III)	2 (0.3)
Benson Figure	2 (0.3)
Cambridge Neurological Test Automated Battery (CANTAB)	2 (0.3)
Card rotations test	2 (0.3)
Cognitive Activity questionnaire	2 (0.3)

For peer review only

Digit vigilance	2 (0.3)
Fluid intelligence	2 (0.3)
Forward and reverse memory span	2 (0.3)
Identical pictures test	2 (0.3)
Lifetime Experience Questionnaire	2 (0.3)
List sorting working memory (LSWM)	2 (0.3)
NCGG-FAT (National Center for Geriatrics and Gerontology Functional Assessment Tool)	2 (0.3)
Neuro-QoL	2 (0.3)
Paper folding task	2 (0.3)
Picture Vocabulary Test (PVT)	2 (0.3)
Preclinical Alzheimer's Cognitive score	2 (0.3)
Prospective memory	2 (0.3)
Story recall	2 (0.3)
Visual Inspection Time	2 (0.3)
Tower of London	2 (0.3)

Biological

A hundred studies (13.8%) utilised biological sampling from serum or whole blood, cerebrospinal fluid, or post-mortem brain tissue to measure brain health (Table 3). ApoE4 genotyping was the most common brain health measurement method in this category, used in 5.6% of all studies in our review. Other commonly measured biomarkers included brain-derived neurotrophic factor (BDNF), neurofilament light from cerebrospinal fluid and tau protein levels.

Table 3: Biological methods for brain health measurement

**Measurement method**	**Number of studies using this method (%)**
Blood ApoE4 genotype	41 (5.6)
Blood brain-derived neurotrophic factor (BDNF)	33 (4.5)
CSF neurofilament light	11 (1.5)
CSF tau levels	10 (1.4)
Blood Aß 42 or 40	9 (1.2)
Blood tau levels	7 (1.0)
Post-mortem neuropathological evaluation of brain tissue	4 (0.6)
Blood S100Beta levels	3 (0.4)
Blood vascular endothelial growth factor (VEGF)	3 (0.4)
CSF BDNF levels	2 (0.3)
Blood interleukin-8 (IL8) levels	2 (0.3)
Blood glial fibrillary acidic protein (GFAP) levels	2 (0.3)
Blood tumour necrosis factor alpha levels	2 (0.3)
Blood eotaxin levels	2 (0.3)
Blood macrophage inflammatory protein-1alpha levels	2 (0.3)

Clinical

Electroencephalography (EEG) was the most used clinical method of evaluating brain health (3.3% of studies). (Table 4) Several studies employed an EEG derived brain age estimation software to measure brain health. The lifestyle score for brain health (LIBRA), which was a composite score comprising of 12 modifiable risk factors for dementia was used in 1.7% of studies. Clinical diagnosis of dementia and hand grip strength were also used as indicators of brain health.

For peer review only

Table 4: Clinical methods for brain health measurement

Measurement method	Number of studies using this method (%)
Electroencephalography (EEG)	24 (3.3)
Lifestyle for brain health (LIBRA) index	12 (1.7)
EEG based brain age	5 (0.7)
Mindreader (EEG software)	4 (0.6)
Clinical diagnosis of Alzheimer's disease or dementia	4 (0.6)
Hand grip strength	4 (0.6)
Test of premorbid functioning	2 (0.3)

Mental health

Thirty-seven (5.1%) studies measured mental health outcomes as an indicator of brain health. (Table 5) Impulsiveness, depression, anxiety, stress, sleep quality, health-related quality of life, and trauma were all behaviours or mental health conditions screened, using a variety of tools.

Table 5: Mental health methods for brain health measurement

Measurement method	Number of studies using this method (%)
Barratt impulsiveness scale	11 (1.5)
Geriatric depression scale	7 (1.0)
Patient health questionnaire (PHQ)-9 depression scale	5 (0.7)
Hospital anxiety and depression (HADS) scales	5 (0.7)
Beck depression inventory	5 (0.7)
Centre for epidemiologic studies depression scale (CES-D)	4 (0.6)
Perceived stress scale	3 (0.4)
Pittsburgh Sleep Quality Index (PSQI)	3 (0.4)
PHQ-8 depression scale	3 (0.4)
Generalised anxiety disorder assessment (GAD)-7	3 (0.4)
Ruminative responses scale	2 (0.3)
RAND-36 health related quality of life survey	2 (0.3)
SF-36 (short form survey)	2 (0.3)
PCL-5 for post-traumatic stress disorder	2 (0.3)

DISCUSSION

Brain health is an emerging research area. Studies about brain health have increased significantly in the last decade, predominantly in the form of cohort studies investigating brain health preservation. Evaluating brain health is complex and there is currently no single test that can be used to fully characterise an individual's brain health. Our scoping review found that brain health is most evaluated via imaging modalities (70.7% of studies) and cognitive testing (45.4% of studies), and approximately a third of all studies used a combination of these two categories of outcomes. Mental health, biological markers and clinical methods of brain health measurement are also used and can provide a more holistic view of an individual's brain health.

Imaging

For peer review only

There are many reasons to use imaging parameters to measure brain health. Magnetic resonance imaging (MRI) enables detailed, non-radioactive, and non-invasive study of the structure, function, and integrity of the brain with minimal risk to participants. Imaging studies can be performed using the same protocol on a large number of participants for cross-sectional comparison and repeated for longitudinal studies (12). It is likely that most studies in our review chose brain volumetric measures to measure brain health due to the ease of obtaining volumetric data from structural MRI scans; the objective, easily interpreted and comparable nature of the data; and existing evidence that brain volume correlates with cognitive performance (13, 14). Structural information only represents the tip of the iceberg of information obtainable through an MRI scan – many scanners have the capability to also perform functional imaging at rest or during tasks; diffusion imaging to explore brain microstructure(15) or other advanced applications such as spectroscopy(16). Each of these modalities provide further information on different aspects of brain health, building a picture of overall brain structure, function, and integrity. Increasing efforts toward collaboration(17, 18) and building large biobanks of brain imaging datasets(19) have led to further innovation with MRI data, where models have been trained to predict brain age using population comparisons(20) or detect and score pathological changes to estimate brain health.

Another benefit of using imaging measurements for brain health include the possibility of focusing on specific regions of the brain, or the presence or absence of specific abnormalities. Dementia is a condition that could be considered the antithesis to brain health, and many studies in our review studied the presence or absence of imaging markers of dementia to estimate brain health. Our findings that the most studied brain regions were the hippocampus and grey matter structures is consistent with the existing literature that these are the regions affected early in Alzheimer's disease (21). Systematic review evidence shows that the presence and increasing volume of white matter hyperintensities (WMH) on MRI are strongly associated with cognitive impairment and all cause dementia (22).

While MRI parameters have been a useful complement in dementia diagnosis, it is widely known that MRI appearances can be heterogeneous even for the same type of dementia and symptom burden (23). Some imaging parameters that appear to be objective, such as fractional anisotropy, are still subject to some degree of human interpretation (to define boundaries, for example) and technical limitations such as the difficulty distinguishing crossing fibres from more structurally robust fibres (24).

Cognitive testing

Neuropsychological and cognitive tests assess a wide range of brain functions, including learning, reading, language and problem-solving skills (25), providing useful insight into a person's cognitive ability. Many tests are cheap or freely available, easy to administer and can be performed in a clinic or home setting without requiring sophisticated equipment, or a prolonged time. Some tests have been adapted into briefer versions or online versions (26), or translated to a different language, further improving their reliability as 'universal' tools (27). The trail making test (TMT) (28), Stroop test (29), Rey auditory verbal learning test (RAVLT) (30), mini-mental state examination (31) and Montreal cognitive assessment (MoCA) (32) were the top five most used cognitive assessments in our review. These are well known, validated tests mainly used as clinical screening tools for cognitive impairment. Several cognitive tests such as the TMT have been adapted to be used during task-based functional MRI scans(28), increasing the ease of combining imaging and cognitive testing when evaluating brain health.

Some limitations of using cognitive testing as the sole method to evaluate brain health include cost, limited sensitivity, ceiling effects and if used repeatedly can lead to bias due to learning. Tests such as the Mini-Mental State Examination (MMSE) incurs a copyright cost of approximately £0.80 (€1.00; $1.30) (2012 data) (33), not including the cost of supervision and test interpretation. Many tests rely on a baseline level of educational qualification or language, meaning that results are unreliable in

For peer review only - http://bmjopen.bmj.com/site/about/guidelines.xhtml

those with a lower educational attainment or those with a different language or cultural background (27). While technological advancements have enabled online or tablet assessments of cognition, problems such as computer anxiety and technological difficulties may limit their generalisability and reproducibility (26). There has yet to be a consensus on a single cognitive test that provides a holistic view of cognitive function, and many studies evaluating and validating cognitive tests have methodological flaws such as small sample sizes, non-generalisable samples, or conflicts of interests.

Biological markers

Biological markers for brain health tend to be objective, quantifiable, and repeatable measures. They can be an efficient method of measuring brain health as it is possible to gain information on various biomarkers using small volumes of blood or cerebrospinal fluid (CSF), and measures can be more easily compared across laboratories if the same protocols are used for sample processing and measurement. *ApoE4*, the most common genetic risk factor for Alzheimer's disease, was the most studied biological marker in our review, likely relating to its use as a predictor for poorer brain health but also its potential as a therapeutic target (34). Brain-derived neurotrophic factor (BDNF), the second most commonly studied biological marker, has been studied as a protective factor and therefore therapeutic target for a wide range of neurological conditions, including those relating to neurodegeneration and mental illness (35). The disadvantages of using biological markers include their invasive nature, problems with sensitivity and specificity and associated cost of performing procedures, advanced laboratory methods, equipment, and interpretation.

Mental health symptom screening

The main advantages of incorporating mental health screening when evaluating brain health include the opportunity for early detection and treatment of common mental health conditions such as depression (36) and anxiety (37); distinguish symptoms due to poor brain health from those relating to poor mental health (38); and promote evidence-based practices that encourage better mental health which can in turn contribute to improving brain health, such as exercise (39) and sleep interventions (40). Challenges researchers may encounter when implementing mental health screening include their self-reported nature, false positives, resource constraints and difficulty performing exhaustive screening for all conditions or selecting specific tests (37). Mental health screening tools suffer from similar methodological issues as cognitive tests – validation is inconsistent across populations, cultures, educational background; some are subject to assessor or performance bias; and there is no single gold standard test available that can measure a person's mental health(41).

Other methods

Other methods of brain health evaluation such as clinical diagnoses, electroencephalography (EEG) and lifestyle or patient-reported brain health scores are all potentially useful methods to measure other aspects of brain health but are all subject to bias and problems with reproducibility, cost, and practical issues.

Strengths and limitations

This is the first review to collate methods of brain health measurement in current literature, providing evidence of rapidly increasing interest in the field over the last decade and identifying the most used brain health outcome measures. This study clearly demonstrates the wide variation in outcome measures and lack of patient-reported outcomes used in brain health research and emphasises the need for outcome set development in this field.

The scoping nature of the review precluded detailed analyses of reasons behind outcome choices and risk of bias in each study. Bias may have been introduced by the lack of standardisation of brain health terminology and definitions during the search and screen, exclusion of non-English language

For peer review only - http://bmjopen.bmj.com/site/about/guidelines.xhtml

papers, and the use of only three databases. The use of several independent reviewers and software reduced the risk of bias during the screening process. The databases and search terms were chosen after extensive consideration and discussion with an independent data specialist within the University of Oxford and the protocol was reviewed by an experienced investigator with extensive experience with Cochrane reviews.

Measurement techniques such as MRI derived volumetric estimations have evolved over the past decade, and new techniques such as machine learning to perform brain age calculations have only recently been developed, limiting the utility of considering frequency of use as the main outcome measure in our review.

CONCLUSIONS AND FUTURE DIRECTIONS

Brain health has become an increasingly popular topic of research and is most frequently evaluated using imaging parameters, alongside other measures such as cognitive testing, biological markers, mental health testing and clinical tests. Future work should focus on fine-tuning brain health definitions and engaging stakeholders and experts to develop a core outcome set for brain health studies that can be informed by findings from this review.

WORD COUNT

3487 words including headings, excluding tables and figures

For peer review only

FUNDING

AL is supported by a clinical doctoral research scholarship from the Nuffield Department of Population Health, University of Oxford. AT is supported by a Wellcome Trust fellowship (216462/Z/19/Z).

CONFLICTS OF INTEREST

The authors declare no conflicts of interest for this project.

AUTHOR CONTRIBUTIONS

AL designed the protocol, led the study, performed the search, first reviewed all abstracts and full texts and extracted data for the work, drafted the manuscript, made edits and submitted the manuscript.

SS, KK, PI, NM, GN, and VP were second reviewers for all abstracts and full texts and extracted data for the work, reviewed the manuscript, and provided final approval for the publication.

AT, RM and NR provided substantial contributions to the conception and design of the study, reviewed the protocol and manuscript, and provided final approval for the publication.

All authors agree to be accountable for all aspects of the work in ensuring that questions related to the accuracy or integrity of any part of the work are appropriately investigated and resolved.

Dr Kapil Savjani provided help with second reviewing for the abstracts and full texts only.

For peer review only

REFERENCES

1. Wang Y, Pan Y, Li H. What is brain health and why is it important? BMJ. 2020;371:m3683.
2. Team WGHE. Ageing. 2019.
3. Global Burden of Disease Study C. Global, regional, and national incidence, prevalence, and years lived with disability for 301 acute and chronic diseases and injuries in 188 countries, 1990-2013: a systematic analysis for the Global Burden of Disease Study 2013. Lancet. 2015;386(9995):743-800.
4. Organisation WH. Brain health 2022 [Available from: https://www.who.int/health-topics/brain-health.
5. Tunis SR, Clarke M, Gorst SL, Gargon E, Blazeby JM, Altman DG, et al. Improving the relevance and consistency of outcomes in comparative effectiveness research. J Comp Eff Res. 2016;5(2):193-205.
6. Qaseem A, Snow V, Cross JT, Jr., Forciea MA, Hopkins R, Jr., Shekelle P, et al. Current pharmacologic treatment of dementia: a clinical practice guideline from the American College of Physicians and the American Academy of Family Physicians. Ann Intern Med. 2008;148(5):370-8.
7. Chalmers I, Glasziou P. Avoidable waste in the production and reporting of research evidence. Obstet Gynecol. 2009;114(6):1341-5.
8. Williamson PR, Barrington H, Blazeby JM, Clarke M, Gargon E, Gorst S, et al. Review finds core outcome set uptake in new studies and systematic reviews needs improvement. J Clin Epidemiol. 2022;150:154-64.
9. Tricco AC, Lillie E, Zarin W, O'Brien KK, Colquhoun H, Levac D, et al. PRISMA Extension for Scoping Reviews (PRISMA-ScR): Checklist and Explanation. Ann Intern Med. 2018;169(7):467-73.
10. Pujol J, Blanco-Hinojo L, Macia D, Martinez-Vilavella G, Deus J, Perez-Sola V, et al. Differences between the child and adult brain in the local functional structure of the cerebral cortex. Neuroimage. 2021;237:118150.
11. Liu X, Gao Y, Di Q, Hu J, Lu C, Nan Y, et al. Differences between child and adult large-scale functional brain networks for reading tasks. Hum Brain Mapp. 2018;39(2):662-79.
12. MacDonald ME, Pike GB. MRI of healthy brain aging: A review. NMR Biomed. 2021;34(9):e4564.
13. Royle NA, Booth T, Valdes Hernandez MC, Penke L, Murray C, Gow AJ, et al. Estimated maximal and current brain volume predict cognitive ability in old age. Neurobiol Aging. 2013;34(12):2726-33.
14. Lovden M, Schmiedek F, Kennedy KM, Rodrigue KM, Lindenberger U, Raz N. Does variability in cognitive performance correlate with frontal brain volume? Neuroimage. 2013;64:209-15.
15. Jones DK, Alexander DC, Bowtell R, Cercignani M, Dell'Acqua F, McHugh DJ, et al. Microstructural imaging of the human brain with a 'super-scanner': 10 key advantages of ultra-strong gradients for diffusion MRI. Neuroimage. 2018;182:8-38.
16. Yousaf T, Dervenoulas G, Politis M. Advances in MRI Methodology. Int Rev Neurobiol. 2018;141:31-76.
17. Niso G, Botvinik-Nezer R, Appelhoff S, De La Vega A, Esteban O, Etzel JA, et al. Open and reproducible neuroimaging: From study inception to publication. Neuroimage. 2022;263:119623.
18. Thompson PM, Jahanshad N, Ching CRK, Salminen LE, Thomopoulos SI, Bright J, et al. ENIGMA and global neuroscience: A decade of large-scale studies of the brain in health and disease across more than 40 countries. Transl Psychiatry. 2020;10(1):100.
19. Littlejohns TJ, Holliday J, Gibson LM, Garratt S, Oesingmann N, Alfaro-Almagro F, et al. The UK Biobank imaging enhancement of 100,000 participants: rationale, data collection, management and future directions. Nat Commun. 2020;11(1):2624.
20. Baecker L, Garcia-Dias R, Vieira S, Scarpazza C, Mechelli A. Machine learning for brain age prediction: Introduction to methods and clinical applications. EBioMedicine. 2021;72:103600.
21. Babcock KR, Page JS, Fallon JR, Webb AE. Adult Hippocampal Neurogenesis in Aging and Alzheimer's Disease. Stem Cell Reports. 2021;16(4):681-93.

For peer review only - http://bmjopen.bmj.com/site/about/guidelines.xhtml

22. Hu HY, Ou YN, Shen XN, Qu Y, Ma YH, Wang ZT, et al. White matter hyperintensities and risks of cognitive impairment and dementia: A systematic review and meta-analysis of 36 prospective studies. Neurosci Biobehav Rev. 2021;120:16-27.
23. Risacher SL, Apostolova LG. Neuroimaging in Dementia. Continuum (Minneap Minn). 2023;29(1):219-54.
24. Figley CR, Uddin MN, Wong K, Kornelsen J, Puig J, Figley TD. Potential Pitfalls of Using Fractional Anisotropy, Axial Diffusivity, and Radial Diffusivity as Biomarkers of Cerebral White Matter Microstructure. Front Neurosci. 2021;15:799576.
25. Eling P. History of Neuropsychological Assessment. Front Neurol Neurosci. 2019;44:164-78.
26. Zygouris S, Tsolaki M. Computerized cognitive testing for older adults: a review. Am J Alzheimers Dis Other Demen. 2015;30(1):13-28.
27. Chun CT, Seward K, Patterson A, Melton A, MacDonald-Wicks L. Evaluation of Available Cognitive Tools Used to Measure Mild Cognitive Decline: A Scoping Review. Nutrients. 2021;13(11).
28. Guo Y. A selective review of the ability for variants of the Trail Making Test to assess cognitive impairment. Appl Neuropsychol Adult. 2022;29(6):1634-45.
29. Perianez JA, Lubrini G, Garcia-Gutierrez A, Rios-Lago M. Construct Validity of the Stroop Color-Word Test: Influence of Speed of Visual Search, Verbal Fluency, Working Memory, Cognitive Flexibility, and Conflict Monitoring. Arch Clin Neuropsychol. 2021;36(1):99-111.
30. Ricci M, Graef S, Blundo C, Miller LA. Using the Rey Auditory Verbal Learning Test (RAVLT) to differentiate alzheimer's dementia and behavioural variant fronto-temporal dementia. Clin Neuropsychol. 2012;26(6):926-41.
31. Arevalo-Rodriguez I, Smailagic N, Roque IFM, Ciapponi A, Sanchez-Perez E, Giannakou A, et al. Mini-Mental State Examination (MMSE) for the detection of Alzheimer's disease and other dementias in people with mild cognitive impairment (MCI). Cochrane Database Syst Rev. 2015;2015(3):CD010783.
32. Hobson J. The Montreal Cognitive Assessment (MoCA). Occup Med (Lond). 2015;65(9):764-5.
33. Seshadri M, Mazi-Kotwal N. A copyright-free alternative to the mini-mental state examination is needed. BMJ. 2012;345:e8589.
34. Safieh M, Korczyn AD, Michaelson DM. ApoE4: an emerging therapeutic target for Alzheimer's disease. BMC Med. 2019;17(1):64.
35. Colucci-D'Amato L, Speranza L, Volpicelli F. Neurotrophic Factor BDNF, Physiological Functions and Therapeutic Potential in Depression, Neurodegeneration and Brain Cancer. Int J Mol Sci. 2020;21(20).
36. Halfin A. Depression: the benefits of early and appropriate treatment. Am J Manag Care. 2007;13(4 Suppl):S92-7.
37. Stein MB, Hill LL. Are There Reasons to Fear Anxiety Screening? JAMA. 2023;329(24):2132-4.
38. Byrne GJ, Pachana NA. Anxiety and depression in the elderly: do we know any more? Curr Opin Psychiatry. 2010;23(6):504-9.
39. Smith PJ, Merwin RM. The Role of Exercise in Management of Mental Health Disorders: An Integrative Review. Annu Rev Med. 2021;72:45-62.
40. Scott AJ, Webb TL, Martyn-St James M, Rowse G, Weich S. Improving sleep quality leads to better mental health: A meta-analysis of randomised controlled trials. Sleep Med Rev. 2021;60:101556.
41. Fusar-Poli P, Salazar de Pablo G, De Micheli A, Nieman DH, Correll CU, Kessing LV, et al. What is good mental health? A scoping review. Eur Neuropsychopharmacol. 2020;31:33-46.

For peer review only

APPENDICES
APPENDIX I: SEARCH STRATEGY

("brain health"(TextWord) AND Measurement terms (All Fields))

OR

((("Cerebrospinal fluid" OR "diagnostic imaging" OR anisotropy OR apolipoprotein OR apolipoproteins OR "arterial spin labelling" OR "brain derived neurotrophic factor" OR biomarker OR biomarkers OR "blood oxygen level dependent" OR "brain function" OR "brain imaging" OR "brain volume" OR "brain tissue volume" OR "cognitive assessment" OR "cognitive interview" OR "cognitive interviews" OR "DNA methylation" OR "genome wide association study" OR GWAS OR "gray matter" OR "grip strength" OR "hand strength" OR "hippocampal volume" OR "magnetic resonance imaging" OR MRI OR "magnetic resonance spectroscopy" OR "mental health statistics" OR "mental status and dementia tests" OR "mini mental state exam" OR "mini mental state examination" OR MMSE OR "motor control" OR "neurocognitive function" OR neuroimaging OR "neuropsychological tests" OR "neuropsychological testing" OR "non verbal reasoning" OR "nonverbal reasoning" OR "nutritional status" OR "physical function" OR "positron emission tomography" OR proteomic OR proteomics OR "psychological tests" OR "psychological testing" OR "self report" OR "short sleep duration" OR "surveys and questionnaires" OR "computed tomography" OR "voxel based morphometry" OR "white matter hyperintensities")) AND ((brain[Title] OR "white matter"[Title] OR Brain[MeSH Terms]) AND (atroph*[Title] OR Alzheimer*[Title] OR cogniti*[Title] OR dementia*[Title] OR "executive function"[Title] OR hippocamp*[Title] OR "mild cognitive impairment"[Title] OR memory[Title] OR "mental disorder"[Title] OR "mental health"[Title] OR neurocognitive[Title] OR neurodegeneration[Title] OR "postoperative cognitive"[Title] OR stroke*[Title] OR "vascular cognitive"[Title]) AND (health[Title] OR healthy[Title] OR longevity[Title] OR "protective factor*"[Title] OR "quality of life"[Title] OR psycholog*[Title] OR resilience[Title] OR well-being[Title] OR health[MeSH Terms] OR outcome assessment health care[MeSH Terms] OR quality of life[MeSH Terms] OR psychological well-being[MeSH Terms]))) OR (("brain health"[Text Word]) AND (("Cerebrospinal fluid" OR "diagnostic imaging" OR anisotropy OR apolipoprotein OR apolipoproteins OR "arterial spin labelling" OR "brain derived neurotrophic factor" OR biomarker OR biomarkers OR "blood oxygen level dependent" OR "brain function" OR "brain imaging" OR "brain volume" OR "brain tissue volume" OR "cognitive assessment" OR "cognitive interview" OR "cognitive interviews" OR "DNA methylation" OR "genome wide association study" OR GWAS OR "gray matter" OR "grip strength" OR "hand strength" OR "hippocampal volume" OR "magnetic resonance imaging" OR MRI OR "magnetic resonance spectroscopy" OR "mental health statistics" OR "mental status and dementia tests" OR "mini mental state exam" OR "mini mental state examination" OR MMSE OR "motor control" OR "neurocognitive function" OR neuroimaging OR "neuropsychological tests" OR "neuropsychological testing" OR "non verbal reasoning" OR "nonverbal reasoning" OR "nutritional status" OR "physical function" OR "positron emission tomography" OR proteomic OR proteomics OR "psychological tests" OR "psychological testing" OR "self report" OR "short sleep duration" OR "surveys and questionnaires" OR "computed tomography" OR "voxel based morphometry" OR "white matter hyperintensities")))

Search limits: English language, Human

For peer review only

APPENDIX II: PRISMA DIAGRAM

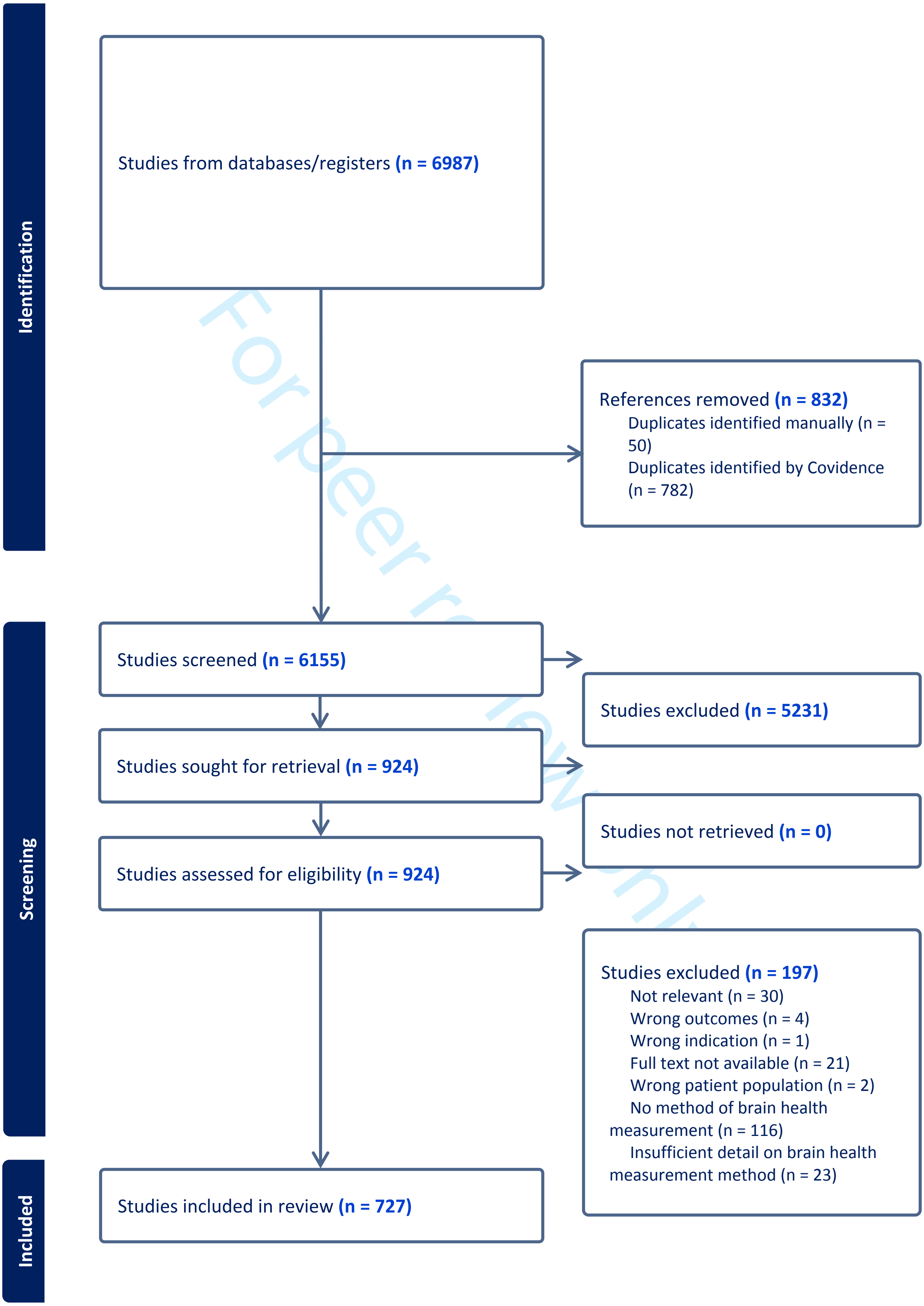

For peer review only - http://bmjopen.bmj.com/site/about/guidelines.xhtml

APPENDIX III: DATA EXTRACTION INSTRUMENT

All numbered items were presented as checkboxes for reviewers to tick for all that applied for each study.

Study characteristics

Author last name [whitespace]

Year of publication[whitespace]

Type of study

1. Randomised controlled trial
2. Cohort study
3. Systematic review/meta-analysis
4. Narrative review
5. Case series
6. Other

Any specific definition of brain health listed in paper? (optional) [whitespace]

Methods of brain health measurement

Imaging

Structural MRI

1. Total/whole brain volume
2. Total/whole brain gray matter volume
3. Total/whole brain white matter volume
4. Hippocampal volume
5. Gray matter volume in specific region(s)
6. White matter volume in specific region(s)
7. Cerebrospinal fluid (CSF) volume in specific region(s)
8. White matter hyperintensities (WMHs)
9. Cerebral microbleeds (CMBs)
10. Cortical thickness
11. White matter lesion volume

Functional MRI

1. Resting state functional connectivity
2. Task based functional connectivity
3. Cerebral blood flow (arterial spin labelling/BOLD responses)
4. Cerebral blood volume
5. Cerebral oxygen consumption
6. Cerebral metabolic rate of oxygen (CMRO2)

Diffusion MRI

For peer review only - http://bmjopen.bmj.com/site/about/guidelines.xhtml

1. Mean diffusivity
2. Axial diffusivity
3. Radial diffusivity
4. Fractional anisotropy
5. Fibre bundle lengths

Compound imaging

1. Brain age estimations (image based)
2. Brain age gap calculations
3. Brain atrophy and lesion index (BALI)
4. Brain health quotients (BHQ)

PET scans/ Spectroscopy/Misc

1. Amyloid load/status
2. Lactate
3. N-acetylaspartate
4. Glutamate
5. Glutamine
6. Magnetoencephalography (MEG) - functional connectivity

Other imaging parameters not previously specified[whitespace]

Genetics/blood/CSF/other

Genetics

1. ApoE4 allele/gene variation

Blood/CSF biomarkers

1. BDNF (serum/blood)
2. BDNF (CSF)
3. Tau (blood)
4. Tau (CSF)
5. Abeta42 or 40 (blood)
6. Abeta 42 or 40 (CSF)
7. IL8 (blood)
8. IL8 (CSF)
9. ACE angiotensin converting enzyme (blood)

EEG

1. Mindreader
2. EEG based brain age
3. EEG unspecified

Clinical

For peer review only

1. Hand grip strength
2. Clinical diagnosis of alzheimer's disease/dementia
3. Oculometric test
4. Duplex ultrasonography of carotids
5. Transcranial doppler ultrasound
6. Transcranial magnetic stimulation (TMS)

Mental health

1. Hospital anxiety and depression (HADS) scales
2. Centre for epidemiologic studies depression scale (CES-D)
3. Beck anxiety inventory
4. Beck depression inventory-ii
5. Pittsburgh Sleep Quality index
6. Mayo clinic fluctuations scale
7. Quality of life scale
8. Zung self rating depression scale
9. Self reported mental health history
10. Apathy scale

Combination scores

1. Brain health score (lifestyle based)
2. Brain health test (BHT)
3. Lifestyle for brain health (LIBRA) index
4. Resilience index

Any other clinical/mental health/combo scores/genetic measures not previously reported[whitespace]

Cognitive tests

Cognitive tests (A-Z)

1. Mini-mental status examination (MMSE)
2. Montreal cognitive assessment (MoCA)
3. Activities specific balance confidence scale
4. Adulthood cognitive activity questionnaire
5. Alzheimer's disease assessment cale (ADAS-cog)
6. Animal naming
7. Apathy inventory
8. Attention network test (ANT)
9. Auditory consonant trigrams
10. Awareness of social inference test (TASIT-R)
11. Behaviour rating inventory of executive function adult version (BRIEF-A)
12. Behavioural assessment of dysexecutive syndrome (BADS)
13. Bell cancellation test
14. Benson Figure Recall
15. Benton Visual Retention Test (BVRT)
16. Boston diagnostic aphasia exam
17. Boston naming test (BNT)

For peer review only

18. Brief cognitive ability measure (B-CAM)
19. Brief visuospatial memory test-revised (BVMT)
20. Buschke selective reminding test
21. California verbal learning test (CVLT-II)
22. Cambridge Cognition Paired Associates Learning
23. CANTAB intra extra dimensional set shift (IED)
24. Category fluency
25. CERAD word list delayed recall and memory battery
26. Clinical dementia rating (CDR)
27. Clock drawing test (CDT)
28. CNS vital signs
29. Cogniciti's brain health assessment (BHA)
30. Cogstate brief battery (CBB)
31. Cogstate neurocognitive battery
32. Cohen's relational memory
33. Color trails test
34. Color word interference test (CWIT)
35. COMPASS-ND neuropsychological battery
36. Continuous paired associative learning (CPAL) task
37. Controlled oral word association test
38. Corsi block test
39. Delis-Kaplan executive functioning (D-KEFS)
40. Digit span
41. Digit symbol substitution test (DSST)
42. Direct assessment of functional status revised (DAFS-R)
43. DKEFS Color Word Intereference
44. Edinburgh Handedness Inventory (EHI)
45. Everyday cognition scale (ECog)
46. Face name association test
47. Flanker NIH Toolbox
48. Frontal assessment battery (FAB)
49. Frontal systems behaviour scale (FrSBe)
50. Geriatric anxiety inventory
51. Gerontology functional assessment tool (NCGG-FAT)
52. Grooved pegboard (PEGS)
53. Hasegawa's dementia scale-revised (HDS-R)
54. Hooper visual organisation test
55. Hopkins verbal learning test (HVLT)
56. Identical pairs version
57. Illness intrusiveness rating scale (IIRS)
58. Inspection time task
59. Iowa Gambling Task
60. IQ test
61. Letter fluency
62. Letter number sequencing
63. Lifetime of experiences questionnaire (LEQ)
64. Logical memory
65. MATRICS consensus cognitive battery (MCCB)
66. Mattis dementia rating scale (DRS)
67. Maryo clinic study of ageinig (MCSA)
68. Memory tool box task

For peer review only

For peer review only - http://bmjopen.bmj.com/site/about/guidelines.xhtml

69. Memtrax memory test
70. MiniCog
71. Mnemonic similarity task (MST)
72. ModBent
73. [Modified] california verbal learning test
74. [Modified] Rey Auditory verbal learning test (RAVLT)
75. Moray House Test No.12
76. MSCEIT managing emotions
77. N- back working memory task
78. NAB mazes
79. National adult reading test
80. National institutes of health (NIH) Toolbox-cognitive battery
81. Neuropsychological assessment scales
82. Number letter computer task
83. Number symbol coding task
84. Pfeffer questionnaire
85. Philadelphia verbal learning test (PVLT)
86. Pittsburgh compound B
87. Positive and negative affect schedule (PANAS)
88. Preclinical Alzheimer's cognitive composite 5 (PACC5)
89. Reaction time tasks
90. Repeatable battery for the assessment of neuropsychological status (RBANS)
91. Rey-Osterrieth Complex Figure test (CFT)
92. RI-48 Cued recall test
93. Set switching
94. Shopping list memory task
95. Short portable mental status questionnaire (SPMSQ)
96. Spatial working memory
97. Stroop color-word test (SCWT)
98. Subjective cognitive decline-questionnaire (SCD-Q)
99. Sustained attention to response task (SART)
100. Symbol digit modalities test (SDMT)
101. TabCAT brain health assessment
102. Task switching tests
103. Technology activities of daily living questionnaire (T-ADLQ)
104. Telephone interview for cognitive status (TICS)
105. Test of everyday attention (TEA)
106. Test of variables of attention (TOVA)
107. The 2 and 7 test
108. Thurstone word fluency test (TWFT)
109. Trail making test (TMT) A or/and B
110. VCAP battery
111. Verbal fluency
112. WAIS-III letter number sequencing
113. Wechsler abbreviated scale of intelligence
114. Wechsler adult intelligence scale (revised)
115. Wechsler memory scale
116. Wechsler test of adult reading
117. Western aphasia battery revised (WAB-AQ)
118. Wide range achievement test (WRAT)
119. Wisconsin Card Sort Test

For peer review only - http://bmjopen.bmj.com/site/about/guidelines.xhtml

Additional cognitive tests not previously mentioned[whitespace]

For peer review only

For peer review only - http://bmjopen.bmj.com/site/about/guidelines.xhtml

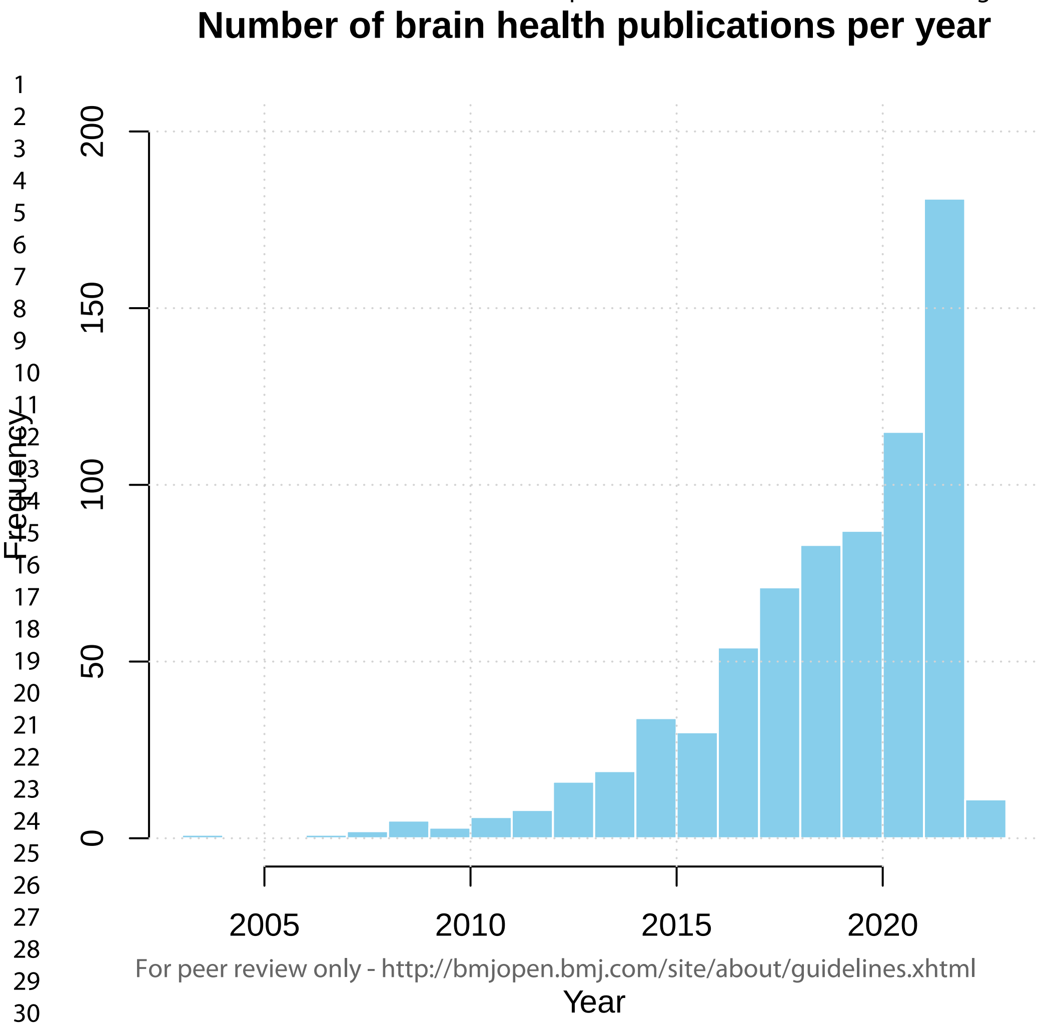
Number of brain health publications per year
Frequency
0
50
100
150
200
2005
2010
2015
2020
Year

Preferred Reporting Items for Systematic reviews and Meta-Analyses extension for Scoping Reviews (PRISMA-ScR) Checklist

SECTION	ITEM	PRISMA-ScR CHECKLIST ITEM	REPORTED ON PAGE #
**TITLE**			
Title	1	Identify the report as a scoping review.	
**ABSTRACT**			
Structured summary	2	Provide a structured summary that includes (as applicable): background, objectives, eligibility criteria, sources of evidence, charting methods, results, and conclusions that relate to the review questions and objectives.	
**INTRODUCTION**			
Rationale	3	Describe the rationale for the review in the context of what is already known. Explain why the review questions/objectives lend themselves to a scoping review approach.	
Objectives	4	Provide an explicit statement of the questions and objectives being addressed with reference to their key elements (e.g., population or participants, concepts, and context) or other relevant key elements used to conceptualize the review questions and/or objectives.	
**METHODS**			
Protocol and registration	5	Indicate whether a review protocol exists; state if and where it can be accessed (e.g., a Web address); and if available, provide registration information, including the registration number.	
Eligibility criteria	6	Specify characteristics of the sources of evidence used as eligibility criteria (e.g., years considered, language, and publication status), and provide a rationale.	
Information sources*	7	Describe all information sources in the search (e.g., databases with dates of coverage and contact with authors to identify additional sources), as well as the date the most recent search was executed.	
Search	8	Present the full electronic search strategy for at least 1 database, including any limits used, such that it could be repeated.	
Selection of sources of evidence†	9	State the process for selecting sources of evidence (i.e., screening and eligibility) included in the scoping review.	
Data charting process‡	10	Describe the methods of charting data from the included sources of evidence (e.g., calibrated forms or forms that have been tested by the team before their use, and whether data charting was done independently or in duplicate) and any processes for obtaining and confirming data from investigators.	
Data items	11	List and define all variables for which data were sought and any assumptions and simplifications made.	
Critical appraisal of individual sources of evidence§	12	If done, provide a rationale for conducting a critical appraisal of included sources of evidence; describe the methods used and how this information was used in any data synthesis (if appropriate).	
Synthesis of results	13	Describe the methods of handling and summarizing the data that were charted.	

For peer review only

SECTION	ITEM	PRISMA-ScR CHECKLIST ITEM	REPORTED ON PAGE #
**RESULTS**			
Selection of sources of evidence	14	Give numbers of sources of evidence screened, assessed for eligibility, and included in the review, with reasons for exclusions at each stage, ideally using a flow diagram.	
Characteristics of sources of evidence	15	For each source of evidence, present characteristics for which data were charted and provide the citations.	
Critical appraisal within sources of evidence	16	If done, present data on critical appraisal of included sources of evidence (see item 12).	
Results of individual sources of evidence	17	For each included source of evidence, present the relevant data that were charted that relate to the review questions and objectives.	
Synthesis of results	18	Summarize and/or present the charting results as they relate to the review questions and objectives.	
**DISCUSSION**			
Summary of evidence	19	Summarize the main results (including an overview of concepts, themes, and types of evidence available), link to the review questions and objectives, and consider the relevance to key groups.	
Limitations	20	Discuss the limitations of the scoping review process.	
Conclusions	21	Provide a general interpretation of the results with respect to the review questions and objectives, as well as potential implications and/or next steps.	
**FUNDING**			
Funding	22	Describe sources of funding for the included sources of evidence, as well as sources of funding for the scoping review. Describe the role of the funders of the scoping review.	

JBI = Joanna Briggs Institute; PRISMA-ScR = Preferred Reporting Items for Systematic reviews and Meta-Analyses extension for Scoping Reviews.

* Where *sources of evidence* (see second footnote) are compiled from, such as bibliographic databases, social media platforms, and Web sites.

† A more inclusive/heterogeneous term used to account for the different types of evidence or data sources (e.g., quantitative and/or qualitative research, expert opinion, and policy documents) that may be eligible in a scoping review as opposed to only studies. This is not to be confused with *information sources* (see first footnote).

‡ The frameworks by Arksey and O'Malley (6) and Levac and colleagues (7) and the JBI guidance (4, 5) refer to the process of data extraction in a scoping review as data charting.

§ The process of systematically examining research evidence to assess its validity, results, and relevance before using it to inform a decision. This term is used for items 12 and 19 instead of "risk of bias" (which is more applicable to systematic reviews of interventions) to include and acknowledge the various sources of evidence that may be used in a scoping review (e.g., quantitative and/or qualitative research, expert opinion, and policy document).

From: Tricco AC, Lillie E, Zarin W, O'Brien KK, Colquhoun H, Levac D, et al. PRISMA Extension for Scoping Reviews (PRISMAScR): Checklist and Explanation. Ann Intern Med. 2018;169:467–473. doi: 10.7326/M18-0850.

For peer review only

BMJ Open

Brain health measurement – a scoping review

Journal:	*BMJ Open*
Manuscript ID	bmjopen-2023-080334.R1
Article Type:	Original research
Date Submitted by the Author:	03-Jan-2024
Complete List of Authors:	Lee, Angeline; University of Oxford, Nuffield Department of Population Health<br>Shah, Suraj; Health Education England London<br>Atha, Karyna; University of Oxford<br>Indoe, Peter; Health Education Thames Valley - HETV<br>Mahmoud, Naira; Health Education Thames Valley - HETV<br>Niblett, Guy; Health Education Thames Valley - HETV<br>Pradhan, Vidushi; Health Education Thames Valley - HETV<br>Roberts, Nia; University of Oxford, Bodleian Health Care Libraries,<br>Malouf, Reem; National Perinatal Epidemiology Unit, uffield Department of Population Health<br>Topiwala, Anya; University of Oxford, Nuffield Department of Population Health
**Primary Subject Heading**:	Geriatric medicine
Secondary Subject Heading:	Evidence based practice, Mental health, Neurology, Public health, Radiology and imaging
Keywords:	Dementia, Aging, NEUROLOGY, Old age psychiatry < PSYCHIATRY, PUBLIC HEALTH, Aged, 80 and over

SCHOLARONE™
Manuscripts

I, the Submitting Author has the right to grant and does grant on behalf of all authors of the Work (as defined in the below author licence), an exclusive licence and/or a non-exclusive licence for contributions from authors who are: i) UK Crown employees; ii) where BMJ has agreed a CC-BY licence shall apply, and/or iii) in accordance with the terms applicable for US Federal Government officers or employees acting as part of their official duties; on a worldwide, perpetual, irrevocable, royalty-free basis to BMJ Publishing Group Ltd ("BMJ") its licensees and where the relevant Journal is co-owned by BMJ to the co-owners of the Journal, to publish the Work in this journal and any other BMJ products and to exploit all rights, as set out in our licence.

The Submitting Author accepts and understands that any supply made under these terms is made by BMJ to the Submitting Author unless you are acting as an employee on behalf of your employer or a postgraduate student of an affiliated institution which is paying any applicable article publishing charge ("APC") for Open Access articles. Where the Submitting Author wishes to make the Work available on an Open Access basis (and intends to pay the relevant APC), the terms of reuse of such Open Access shall be governed by a Creative Commons licence – details of these licences and which Creative Commons licence will apply to this Work are set out in our licence referred to above.

Other than as permitted in any relevant BMJ Author's Self Archiving Policies, I confirm this Work has not been accepted for publication elsewhere, is not being considered for publication elsewhere and does not duplicate material already published. I confirm all authors consent to publication of this Work and authorise the granting of this licence.

For peer review only

Brain health measurement – a scoping review

AUTHORS

Angeline Lee[1], Suraj Shah, Karyna Atha[3], Peter Indoe[4], Naira Mahmoud[4], Guy Niblett[4], Vidushi Pradhan[4], Nia Roberts[5], Reem Malouf[1], Anya Topiwala[1]

1. Big Data Institute, Nuffield Department of Population Health, University of Oxford, Oxford, UK
2. South London School of Anaesthesia, Health Education England, London, UK
3. Oxford Medical School, University of Oxford, Oxford, UK
4. Health Education Thames Valley, Oxford, UK
5. Outreach Librarian Knowledge Centre, Bodleian Health Care Libraries, Oxford, UK

Corresponding author:
Dr Angeline Lee, Big Data Institute Level 1 (Nichols group), Nuffield Department of Population Health, University of Oxford, Old Road Campus OX3 7LF
Email: angeline.lee@ndph.ox.ac.uk
Telephone: +447540474162

KEYWORDS

Dementia, Aging, Neurology, Old age psychiatry < Psychiatry, Public Health, Aged, 80 and over

WORD COUNT

4109 words including headings, excluding tables and figures

For peer review only

ABSTRACT

Objectives: Preservation of brain health is an urgent priority for the world's ageing population. The evidence base for brain health optimisation strategies is rapidly expanding, but clear recommendations have been limited by heterogeneity in measurement of brain health outcomes. We performed a scoping review to systematically evaluate brain health measurement in the scientific literature to date, informing development of a core outcome set.

Design: Scoping review

Data sources: Medline, APA PsycArticles and Embase were searched through till 25th January 2023

Eligibility criteria for selecting studies: Studies were included if they described brain health evaluation methods in sufficient detail in human adults and were in English language.

Data extraction and synthesis: Two reviewers independently screened titles, abstracts and full texts for inclusion and extracted data using Covidence software.

Results: From 6987 articles identified by the search, 727 studies met inclusion criteria. Study publication increased by 22 times in the last decade. Cohort study was the most common study design (n=609,84%). 479 unique methods of measuring brain health were identified, comprising imaging, cognitive, mental health, biological and clinical categories. Seven of the top ten most frequently used brain health measurement methods were imaging-based, including structural imaging of grey matter and hippocampal volumes and white matter hyperintensities. Cognitive tests such as the trail making test accounted for 286 (59.7%) of all brain health measurement methods.

Conclusions: The scientific literature surrounding brain health has increased exponentially, yet measurement methods are highly heterogeneous across studies which may explain lack clinical translation. Future studies should aim to develop a selected group of measures that should be included in all brain health studies to aid inter-study comparison (core outcome set); and broaden from the current focus on neuroimaging outcomes to include a range of outcomes.

STRENGTHS AND LIMITATIONS OF THIS STUDY

- Broad search strategy developed after a preliminary search of the current evidence base
- Wide inclusion criteria to capture maximal number of relevant studies
- Protocol does not include description of risk of bias for included studies
- Non-English language articles were excluded

For peer review only

INTRODUCTION

Brain health can be defined as the preservation of optimal brain integrity and mental and cognitive function at a given age in the absence of overt brain diseases that affect normal brain function.(1) The ageing population in the world is increasing and the number of people aged over 60 is expected to grow to 2 billion in 2050 (2).

The Global Burden of Disease study 2013 demonstrated that neurological disorders are a leading cause of chronic disorders worldwide, and that the years lived with disability for all neurological disorders increased by 59.6% from 1990 to 2013 as people are living for longer. The years lived with disability for Alzheimer's disease alone increased by 91.8% from 1990 to 2013 (3). Ten years on, the burden of disease has increased even further. In May 2022, the World Health Organisation member states implemented a global action plan to improve healthcare and wellbeing of people living with neurological disorders and reducing mortality, morbidity and disability associated with these conditions (4).

The time is ripe to invest in methods of improving and optimising brain health to maximise the population quality of life and minimise disability, disease and death related to neurological diseases (1).

The research world has responded by launching many studies to trial interventions to preserve brain health, but the wide variation in the methods used to study brain health is limiting comparison between studies (5) and therefore recommendations for interventions that can potentially improve brain health (6). This has led to wasteful research practices – including repetition of studies comparing similar interventions but measuring different outcomes (5, 7). There is no consensus on a set of brain health outcomes that would be meaningful and important to patients, nor is there one on how specific outcomes should be measured and reported. There is an urgent need to achieve a consensus in brain health reporting to encourage prevention, optimisation and potentially even treatment for neurological diseases.

We aimed to conduct a systematic scoping review to evaluate methods of brain health measurement in current literature. This would enable us to identify and group brain health measurement tools and evaluate patterns of use of specific tools based on study locations, study types and year of publication. Core outcome sets (COS) are agreed standardised sets of outcomes that should be measured and reported, as a minimum, in all clinical trials in specific areas of health or healthcare(5). These could be extended to include other types of study design. Despite the introduction of organisations such as the Core Outcome Measures in Effectiveness Trials (COMET) initiative in 2010 and support from various organisations to boost COS use in research, COS uptake is low in many branches of research including brain health(8). This scoping review can provide a useful overview of the current state of brain health research and provide a list of tools for brain health measurement that can be considered in COS development.

A scoping review was chosen as the best technique to perform an initial rapid mapping of current evidence on brain health and identify the most used brain health outcome measures, to inform future consensus work on brain health outcomes to facilitate development of a brain health COS.

METHODS

This scoping review was conducted in accordance with the Preferred Reporting Items for Systematic reviews and meta-analyses extension for scoping reviews (PRISMA-ScR) checklist (9).

For peer review only - http://bmjopen.bmj.com/site/about/guidelines.xhtml

A preliminary search of Medline, the Cochrane Database of Systematic Reviews and JBI Evidence Synthesis was conducted, and no current or underway systematic reviews or scoping reviews were identified on this topic. Over 3000 papers were found on the preliminary Medline search with the search terms ("brain-health" OR “cognitive-health”) AND ("measur*" OR "outcome*" OR "biomarker" OR "marker"), so there was sufficient evidence available to inform this review.

Inclusion/exclusion criteria

Studies that met the following criteria were included in the review:

1. Participants must be human
2. Participants must be aged 18 years or over
3. Studies must report outcomes that are measuring ‘brain health’
4. Studies must be written in the English language

Studies were excluded from the review if they did not report brain health measures with sufficient detail to enable replication, for example studies that reported that imaging was used without specifying fractional anisotropy as the measurement.

The human brain develops significantly between childhood and adulthood, with different structure, network organisation and function (10, 11). Studies about children or adolescents were excluded as brain health measurement tools in children may not be suitable for adults and vice versa. Brain health is a human concept due to the complexity of human brain functions; therefore, we excluded studies on non-humans.

Search strategy

A systematic search was conducted of Medline, Embase and APA PsycArticles databases for articles published from the inception of each of these databases to 25th January 2023 using a search strategy developed with an information specialist (Appendix I). The syntax of the search strategy was modified for use with Embase and APA PsycArticles.

Due to the relatively new concept of brain health, the search strategy was informed by an initial limited search of Medline. The following search terms were used: ("brain-health" OR “cognitive-health”) AND ("measur*" OR "outcome*" OR "biomarker" OR "marker") on 12th December 2022, and 2362 results were screened by one author, of which 72 full text papers were found to be suitable for inclusion for the review. The Yale MeSH analyser was used to extract all MeSH headings and author keywords used in these 72 full text papers. The terms were analysed with Rstudio (Version: 2022.12.0+353 (2022.12.0+353)). 1035 search terms were used in the 72 papers, with 286 distinct search terms. All terms were considered for inclusion into the search strategy (Appendix I).

This scoping review included all study designs, including experimental and quasi-experimental study designs such as randomized controlled trials, non-randomized controlled trials, before and after studies and interrupted time-series studies; analytical observational studies such as prospective and retrospective cohort studies, case-control studies and analytical cross-sectional studies; descriptive observational study designs such as case series, individual case reports and descriptive cross-sectional studies; systematic reviews, text and opinion papers and conference abstracts if they met the inclusion criteria.

Source of evidence selection

For peer review only - http://bmjopen.bmj.com/site/about/guidelines.xhtml

Following the search on 25th January 2023, all identified citations were uploaded into Covidence (Covidence systematic review software, Veritas Health Innovation, Melbourne, Australia. Available at www.covidence.org.), which is a web-based collaboration software platform that streamlines the production of systematic and other literature reviews. Duplicates were removed by Covidence during this process, and further duplicates were manually removed.

Following a pilot test, AL and another independent reviewer (SS, KA, PI, NM, GN, or VP) screened each title and abstract for assessment against the inclusion criteria for the review. Full-text articles for potentially relevant sources were imported into Covidence, and these were assessed in detail against the inclusion criteria by AL and another independent reviewer (SS, KA, PI, NM, GN, or VP). Reasons for exclusion of sources of evidence at full text that did not meet the inclusion criteria were recorded by the system. Conflicts in reviewer opinion were all resolved through discussion, although an additional independent reviewer (AT) was available for adjudication.

The results of the search and the study inclusion process are presented in a Preferred Reporting Items for Systematic Reviews and Meta-analyses extension for scoping review (PRISMA-ScR) flow diagram (9) (Appendix II).

Data extraction

Data was extracted from included papers by two independent reviewers using a data extraction template developed by the reviewers on Covidence (Appendix III). All conflicts were resolved through discussion before the data extraction process was finalised.

All brain health measurement methods were grouped into categories and tabulated based on frequency of use. Study location was determined from the methods section of each study, and if this was not mentioned or an international cohort was used, the country of the first author's institution was entered as the study location. Study location was not entered for narrative or systematic reviews.

Patient and public involvement

None

RESULTS

A total of 6155 studies were included in the title and abstract screening after removing duplicates from the original search results. After abstract review, 924 studies were assessed for eligibility using the inclusion and exclusion criteria, leaving 727 studies for data extraction (Appendix II).

Study types

There were 609 (83.8%) cohort studies, 59 (8.1%) randomised controlled trials or sub studies within randomised controlled trials; 25 (3.4%) case series; 19 (2.6%) systematic reviews with or without meta-analyses; 11 (1.5%) narrative reviews and 4 (0.6%) were other study types.

For peer review only

There was a wide heterogeneity in brain health measurement methods between study types, and more than 60% of studies in each type utilised more than one modality (imaging, cognitive, mental health, clinical or biological) to measure brain health (Supplemental Table 1). Mental health measurement methods were the least used category, used in only 11 cohort studies and 2 narrative reviews.

Temporal trends

The range of years of publication of brain health studies was between year 2003 and 2023. Supplementary figure A shows a histogram of number of brain health publications per year. The number of published brain health studies is steadily increasing and has more than tripled in the last 5 years (54 papers published in 2017 and 181 papers published in 2022), and 22 times in the last 10 years (8 papers published in 2012).

The percentage of studies utilising mental health, clinical and biological methods to measure brain health has increased in the last five years, and the number of studies using multiple categories of brain health measurement has increased over time (Supplemental Table 2).

Geographic trends

Supplementary figure B shows a heat map of the study location of the 697 brain health publications from this review (this data excludes systematic and narrative reviews). Supplemental Table 3 shows a list of the 39 countries where brain health studies were carried out and the number of studies utilising each category of brain health measurement. The United States (US) alone accounts for almost 50% of all published brain health studies, with most studies published in the states of California, Massachusetts, and Maryland (22, 10, and 8% of all US studies respectively). The top five countries researching brain health (in order) were the US, United Kingdom, Canada, Australia, and China. Only 43 studies (6% of 697) took place in eleven low- or middle-income countries (LMIC) defined by the World Bank, and the two studies that took place on the African continent were led by US or UK researchers. Half the studies in LMICs were multicategory studies, and the other half used imaging techniques as the sole method of brain health measurement. None of the LMIC studies used mental health or biological techniques to measure brain health.

Brain health measurement methods

There were 478 unique methods of brain health measurement identified in the data extraction. Two hundred and sixty-eight (56.1%) of these were only used once. The remaining 210 methods will be presented in imaging, biological, clinical, mental health, and cognitive test categories.

Within these categories, 1 study (0.1%) included outcome measures from 4 categories (cognitive, mental health, clinical and biological); 34 studies (4.7%) included measures from 3 categories (most commonly imaging, cognitive, and biological); 233 studies (32.0%) included measures from 2 categories (most commonly imaging and cognitive); and the remaining 460 studies (63.3%) included measures from one of these categories (most commonly imaging).

Eight of the top ten most prevalent methods for measuring brain health were imaging based (Table 1). These were mainly volume estimates for gray and white matter in specific regions, particularly the hippocampus, and the whole brain; presence of white matter hyperintensities, and fractional anisotropy. The trail making test and mini-mental status examination (MMSE) were the other two most prevalent methods (Table 1).

For peer review only - http://bmjopen.bmj.com/site/about/guidelines.xhtml

Table 1: Top ten most used measures for brain health measurements

Measurement method	Category	Number of studies using this method (%)
Gray matter volume in specific region(s)	Imaging	133 (18.3)
White matter hyperintensities (WMHs)	Imaging	133 (18.3)
Total brain volume	Imaging	132 (18.2)
Whole brain gray matter volume	Imaging	106 (14.6)
Hippocampal volume	Imaging	105 (14.4)
Fractional anisotropy	Imaging	102 (14.0)
White matter volume in specific region(s)	Imaging	95 (13.1)
Trail making test (TMT) A and/or B	Cognitive testing	86 (11.8)
Whole brain white matter volume	Imaging	77 (10.6)
Mini-mental status examination (MMSE)	Cognitive testing	73 (10.0)

*Row data is not mutually exclusive as many studies used more than 1 category of methods

Imaging

Imaging was the most common method of brain health measurement (514 studies, 70.7%), particularly magnetic resonance imaging (MRI) based measures. Within imaging, measurements were divided into structural, functional, diffusion MRI parameters, compound imaging indices, and miscellaneous forms of imaging (Supplemental Table 4).

Approximately a fifth of all studies in our review utilised structural MRI based volumetric estimates, particularly of grey matter and hippocampal volumes; or looked for the presence of white matter hyperintensities. Seven percent of studies looked at cerebral blood flow in specific regions of the brain using functional MRI techniques at rest or whilst performing tasks. Brain age gap calculations comparing an imaging estimate of brain age derived from various MRI parameters to a person's chronological age were used in 1.8% of studies. Positron emission tomography (PET) measured amyloid load or presence was the most used (5.2%) type of non-MRI imaging method to measure brain health.

Cognitive tests

Three-hundred and thirty (45.4%) studies used a form of cognitive test when measuring brain health. The highest number of individual brain health measurement methods used more than once were in this category (115/210, 54.8%). Only named test batteries or tests described in sufficient detail for replication were included in the data extraction.

The trail making test A or B, mini-mental status examination and Stroop tests were the most used of all cognitive tests, with approximately a tenth of all studies using one or more of these in evaluating brain health (Supplemental Table 5).

Biological

A hundred studies (13.8%) utilised biological sampling from serum or whole blood, cerebrospinal fluid, or post-mortem brain tissue to measure brain health (Supplemental Table 6). ApoE4 genotyping was the most common brain health measurement method in this category, used in 5.6% of all studies

For peer review only - http://bmjopen.bmj.com/site/about/guidelines.xhtml

in our review. Other commonly measured biomarkers included brain-derived neurotrophic factor (BDNF), neurofilament light from cerebrospinal fluid and tau protein levels.

Clinical

Electroencephalography (EEG) was the most used clinical method of evaluating brain health (3.3% of studies) (Supplemental Table 7). Several studies employed an EEG derived brain age estimation software to measure brain health. The lifestyle score for brain health (LIBRA), which was a composite score comprising of 12 modifiable risk factors for dementia was used in 1.7% of studies. Clinical diagnosis of dementia and hand grip strength were also used as indicators of brain health. Sleep quality indexes and health related quality of life surveys were also included in this category.

Mental health

Thirty-seven (5.1%) studies measured mental health outcomes as an indicator of brain health (Supplemental Table 8). The most commonly used measure was the Baratt impulsiveness scale, followed by a number of screening tools for depressive and anxiety symptoms. Other, more rarely used measures included those designed to identify perceived stress, rumination, and post-traumatic stress disorder symptoms.

DISCUSSION

Brain health is an emerging research area. Studies about brain health have increased significantly in the last decade, predominantly in the form of cohort studies investigating brain health preservation. Evaluating brain health is complex and there is currently no single test that can be used to fully characterise an individual's brain health. Our scoping review found that brain health is most evaluated via imaging modalities (70.7% of studies) and cognitive testing (45.4% of studies), and approximately a third of all studies used a combination of these two categories of outcomes. Mental health, biological markers and clinical methods of brain health measurement are also used and can provide a more holistic view of an individual's brain health.

Imaging

There are many reasons to use imaging parameters to measure brain health. Magnetic resonance imaging (MRI) enables detailed, non-radioactive, and non-invasive study of the structure, function, and integrity of the brain with minimal risk to participants. Imaging studies can be performed using the same protocol on a large number of participants for cross-sectional comparison and repeated for longitudinal studies (12). It is likely that most studies in our review chose brain volumetric measures to measure brain health due to the ease of obtaining volumetric data from structural MRI scans; the objective, easily interpreted and comparable nature of the data; and existing evidence that brain volume correlates with cognitive performance (13, 14). Structural information only represents the tip of the iceberg of information obtainable through an MRI scan – many scanners have the capability to also perform functional imaging at rest or during tasks; diffusion imaging to explore brain microstructure(15) or other advanced applications such as spectroscopy(16). Each of these modalities provide further information on different aspects of brain health, building a picture of overall brain structure, function, and integrity. Increasing efforts toward collaboration(17, 18) and building large biobanks of brain imaging datasets(19) have led to further innovation with MRI data, where models have been trained to predict brain age using population comparisons(20) or detect and score pathological changes to estimate brain health.

For peer review only

Another benefit of using imaging measurements for brain health include the possibility of focusing on specific regions of the brain, or the presence or absence of specific abnormalities. Dementia is a condition that could be considered the antithesis to brain health, and many studies in our review studied the presence or absence of imaging markers of dementia to estimate brain health. Our findings that the most studied brain regions were the hippocampus and grey matter structures is consistent with the existing literature that these are the regions affected early in Alzheimer's disease (21). Systematic review evidence shows that the presence and increasing volume of white matter hyperintensities (WMH) on MRI are strongly associated with cognitive impairment and all cause dementia (22).

While MRI parameters have been a useful complement in dementia diagnosis, it is widely known that MRI appearances can be heterogeneous even for the same type of dementia and symptom burden (23). Some imaging parameters that appear to be objective, such as fractional anisotropy, are still subject to some degree of human interpretation (to define boundaries, for example) and technical limitations such as the difficulty distinguishing crossing fibres from more structurally robust fibres (24).

Another drawback of using MRI as a key method of measuring brain health is its significant cost. The United Kingdom's national health service (NHS) estimates a cost per unit of MRI scan of one location without contrast as £146.75(25) (€170.51; $183.87), and another study estimated a diffusion weighted MRI scan of the brain for cholesteatoma patients to be in the region of $390.66 Canadian dollars (€266.63, $287.54)(26). These costs do not consider the cost of setup or maintenance of an MRI machine, or the potential need for specialist staff to run specific imaging protocols or interpret images from different modalities. The small number of brain health studies from low to middle income countries may reflect difficulties in funding brain health research.

Cognitive testing

Neuropsychological and cognitive tests assess a wide range of brain functions, including learning, reading, language and problem-solving skills (27), providing useful insight into a person's cognitive ability. Many tests are cheap or freely available, easy to administer and can be performed in a clinic or home setting without requiring sophisticated equipment, or a prolonged time. Some tests have been adapted into briefer versions or online versions (28), or translated to a different language, further improving their reliability as 'universal' tools (29). The trail making test (TMT) (30), Stroop test (31), Rey auditory verbal learning test (RAVLT) (32), mini-mental state examination (33) and Montreal cognitive assessment (MoCA) (34) were the top five most used cognitive assessments in our review. These are well known, validated tests mainly used as clinical screening tools for cognitive impairment. Several cognitive tests such as the TMT have been adapted to be used during task-based functional MRI scans(30), increasing the ease of combining imaging and cognitive testing when evaluating brain health.

Some limitations of using cognitive testing as the sole method to evaluate brain health include cost, limited sensitivity, ceiling effects and if used repeatedly can lead to bias due to learning. Tests such as the Mini-Mental State Examination (MMSE) incurs a copyright cost of approximately £0.80 (€1.00; $1.30) (2012 data) (35), not including the cost of supervision and test interpretation. Many tests rely on a baseline level of educational qualification or language, meaning that results are unreliable in those with a lower educational attainment or those with a different language or cultural background (29). While technological advancements have enabled online or tablet assessments of cognition, problems such as computer anxiety and technological difficulties may limit their generalisability and reproducibility (28). There has yet to be a consensus on a single cognitive test that provides a holistic view of cognitive function, and many studies evaluating and validating cognitive tests have methodological flaws such as small sample sizes, non-generalisable samples, or conflicts of interests.

Biological markers

For peer review only - http://bmjopen.bmj.com/site/about/guidelines.xhtml

Biological markers for brain health tend to be objective, quantifiable, and repeatable measures. They can be an efficient method of measuring brain health as it is possible to gain information on various biomarkers using small volumes of blood or cerebrospinal fluid (CSF), and measures can be more easily compared across laboratories if the same protocols are used for sample processing and measurement. *ApoE4*, the most common genetic risk factor for Alzheimer's disease, was the most studied biological marker in our review, likely relating to its use as a predictor for poorer brain health but also its potential as a therapeutic target (36). Brain-derived neurotrophic factor (BDNF), the second most commonly studied biological marker, has been studied as a protective factor and therefore therapeutic target for a wide range of neurological conditions, including those relating to neurodegeneration and mental illness (37). The disadvantages of using biological markers include their invasive nature, problems with sensitivity and specificity and associated cost of performing procedures, advanced laboratory methods, equipment, and interpretation.

Mental health symptom screening

The main advantages of incorporating mental health screening when evaluating brain health include the opportunity for early detection and treatment of common mental health conditions such as depression (38) and anxiety (39); distinguish symptoms due to poor brain health from those relating to poor mental health (40); and promote evidence-based practices that encourage better mental health which can in turn contribute to improving brain health, such as exercise (41) and sleep interventions (42). Challenges researchers may encounter when implementing mental health screening include their self-reported nature, false positives, resource constraints and difficulty performing exhaustive screening for all conditions or selecting specific tests (39). Mental health screening tools suffer from similar methodological issues as cognitive tests – validation is inconsistent across populations, cultures, educational background; some are subject to assessor or performance bias; and there is no single gold standard test available that can measure a person's mental health(43).

Other methods

Other methods of brain health evaluation such as clinical diagnoses, electroencephalography (EEG) and lifestyle or patient-reported brain health scores are all potentially useful methods to measure other aspects of brain health but are all subject to bias and problems with reproducibility, cost, and practical issues.

Strengths and limitations

This is the first review to collate methods of brain health measurement in current literature, providing evidence of rapidly increasing interest in the field over the last decade and identifying the most used brain health outcome measures. This study clearly demonstrates the wide variation in outcome measures and lack of patient-reported outcomes used in brain health research and emphasises the need for outcome set development in this field.

The scoping nature of the review precluded detailed analyses of reasons behind outcome choices and risk of bias in each study. Bias may have been introduced by the lack of standardisation of brain health terminology and definitions during the search and screen, exclusion of non-English language papers, and the use of only three databases. The use of several independent reviewers and software reduced the risk of bias during the screening process. The databases and search terms were chosen after extensive consideration and discussion with an independent data specialist within the University of Oxford and the protocol was reviewed by an experienced investigator with extensive experience with Cochrane reviews.

Measurement techniques such as MRI derived volumetric estimations have evolved over the past decade, and new techniques such as machine learning to perform brain age calculations have only

For peer review only - http://bmjopen.bmj.com/site/about/guidelines.xhtml

recently been developed, limiting the utility of considering frequency of use as the main outcome measure in our review.

CONCLUSIONS AND FUTURE DIRECTIONS

Brain health has become an increasingly popular topic of research and is most frequently evaluated using imaging parameters, alongside other measures such as cognitive testing, biological markers, mental health testing and clinical tests.

Future work should focus on fine-tuning brain health definitions and engaging stakeholders and experts to develop a core outcome set (COS) for brain health studies that can be informed by findings from this review. There is an urgent need for a COS in this field to facilitate cross-study comparisons, particularly for interventional studies to improve or maintain brain health. Outcomes should broaden the focus from expensive neuroimaging methods to encompass a more holistic view of the brain, for example mental health outcomes that are currently neglected in the literature. Consensus work involving patients, carers and professionals should be undertaken to ensure the core outcomes are useful and relevant.

For peer review only

Contributorship statement

AL designed the protocol, led the study, performed the search, first reviewed all abstracts and full texts and extracted data for the work, drafted the manuscript, made edits and submitted the manuscript.

SS, KK, PI, NM, GN, and VP were second reviewers for all abstracts and full texts and extracted data for the work, reviewed the manuscript, and provided final approval for the publication.

AT, RM and NR provided substantial contributions to the conception and design of the study, reviewed the protocol and manuscript, and provided final approval for the publication.

All authors agree to be accountable for all aspects of the work in ensuring that questions related to the accuracy or integrity of any part of the work are appropriately investigated and resolved.

Dr Kapil Savjani provided help with second reviewing for the abstracts and full texts.

Competing interests

The authors declare no conflicts of interest for this project.

Funding

AL is supported by a clinical doctoral research scholarship from the Nuffield Department of Population Health, University of Oxford. AT is supported by a Wellcome Trust fellowship (216462/Z/19/Z).

Data sharing statement

No additional data available

For peer review only

REFERENCES

1. Wang Y, Pan Y, Li H. What is brain health and why is it important? BMJ. 2020;371:m3683.
2. World Global Health Ethics Team. Ageing. 2019. [Available from: https://www.who.int/ethics/topics/ageing/en/]
3. Global Burden of Disease Study Consortium. Global, regional, and national incidence, prevalence, and years lived with disability for 301 acute and chronic diseases and injuries in 188 countries, 1990-2013: a systematic analysis for the Global Burden of Disease Study 2013. Lancet. 2015;386(9995):743-800.
4. World Health Organisation. Brain health 2022 [Available from: https://www.who.int/health-topics/brain-health.
5. Tunis SR, Clarke M, Gorst SL, Gargon E, Blazeby JM, Altman DG, et al. Improving the relevance and consistency of outcomes in comparative effectiveness research. J Comp Eff Res. 2016;5(2):193-205.
6. Qaseem A, Snow V, Cross JT, Jr., Forciea MA, Hopkins R, Jr., Shekelle P, et al. Current pharmacologic treatment of dementia: a clinical practice guideline from the American College of Physicians and the American Academy of Family Physicians. Ann Intern Med. 2008;148(5):370-8.
7. Chalmers I, Glasziou P. Avoidable waste in the production and reporting of research evidence. Obstet Gynecol. 2009;114(6):1341-5.
8. Williamson PR, Barrington H, Blazeby JM, Clarke M, Gargon E, Gorst S, et al. Review finds core outcome set uptake in new studies and systematic reviews needs improvement. J Clin Epidemiol. 2022;150:154-64.
9. Tricco AC, Lillie E, Zarin W, O'Brien KK, Colquhoun H, Levac D, et al. PRISMA Extension for Scoping Reviews (PRISMA-ScR): Checklist and Explanation. Ann Intern Med. 2018;169(7):467-73.
10. Pujol J, Blanco-Hinojo L, Macia D, Martinez-Vilavella G, Deus J, Perez-Sola V, et al. Differences between the child and adult brain in the local functional structure of the cerebral cortex. Neuroimage. 2021;237:118150.
11. Liu X, Gao Y, Di Q, Hu J, Lu C, Nan Y, et al. Differences between child and adult large-scale functional brain networks for reading tasks. Hum Brain Mapp. 2018;39(2):662-79.
12. MacDonald ME, Pike GB. MRI of healthy brain aging: A review. NMR Biomed. 2021;34(9):e4564.
13. Royle NA, Booth T, Valdes Hernandez MC, Penke L, Murray C, Gow AJ, et al. Estimated maximal and current brain volume predict cognitive ability in old age. Neurobiol Aging. 2013;34(12):2726-33.
14. Lovden M, Schmiedek F, Kennedy KM, Rodrigue KM, Lindenberger U, Raz N. Does variability in cognitive performance correlate with frontal brain volume? Neuroimage. 2013;64:209-15.
15. Jones DK, Alexander DC, Bowtell R, Cercignani M, Dell'Acqua F, McHugh DJ, et al. Microstructural imaging of the human brain with a 'super-scanner': 10 key advantages of ultra-strong gradients for diffusion MRI. Neuroimage. 2018;182:8-38.
16. Yousaf T, Dervenoulas G, Politis M. Advances in MRI Methodology. Int Rev Neurobiol. 2018;141:31-76.
17. Niso G, Botvinik-Nezer R, Appelhoff S, De La Vega A, Esteban O, Etzel JA, et al. Open and reproducible neuroimaging: From study inception to publication. Neuroimage. 2022;263:119623.
18. Thompson PM, Jahanshad N, Ching CRK, Salminen LE, Thomopoulos SI, Bright J, et al. ENIGMA and global neuroscience: A decade of large-scale studies of the brain in health and disease across more than 40 countries. Transl Psychiatry. 2020;10(1):100.
19. Littlejohns TJ, Holliday J, Gibson LM, Garratt S, Oesingmann N, Alfaro-Almagro F, et al. The UK Biobank imaging enhancement of 100,000 participants: rationale, data collection, management and future directions. Nat Commun. 2020;11(1):2624.
20. Baecker L, Garcia-Dias R, Vieira S, Scarpazza C, Mechelli A. Machine learning for brain age prediction: Introduction to methods and clinical applications. EBioMedicine. 2021;72:103600.

For peer review only

21. Babcock KR, Page JS, Fallon JR, Webb AE. Adult Hippocampal Neurogenesis in Aging and Alzheimer's Disease. Stem Cell Reports. 2021;16(4):681-93.
22. Hu HY, Ou YN, Shen XN, Qu Y, Ma YH, Wang ZT, et al. White matter hyperintensities and risks of cognitive impairment and dementia: A systematic review and meta-analysis of 36 prospective studies. Neurosci Biobehav Rev. 2021;120:16-27.
23. Risacher SL, Apostolova LG. Neuroimaging in Dementia. Continuum (Minneap Minn). 2023;29(1):219-54.
24. Figley CR, Uddin MN, Wong K, Kornelsen J, Puig J, Figley TD. Potential Pitfalls of Using Fractional Anisotropy, Axial Diffusivity, and Radial Diffusivity as Biomarkers of Cerebral White Matter Microstructure. Front Neurosci. 2021;15:799576.
25. (NHS) NHS. National schedule of NHS costs. 2020.
26. Choi DL, Gupta MK, Rebello R, Archibald JD. Cost-comparison analysis of diffusion weighted magnetic resonance imaging (DWMRI) versus second look surgery for the detection of residual and recurrent cholesteatoma. J Otolaryngol Head Neck Surg. 2019;48(1):58.
27. Eling P. History of Neuropsychological Assessment. Front Neurol Neurosci. 2019;44:164-78.
28. Zygouris S, Tsolaki M. Computerized cognitive testing for older adults: a review. Am J Alzheimers Dis Other Demen. 2015;30(1):13-28.
29. Chun CT, Seward K, Patterson A, Melton A, MacDonald-Wicks L. Evaluation of Available Cognitive Tools Used to Measure Mild Cognitive Decline: A Scoping Review. Nutrients. 2021;13(11).
30. Guo Y. A selective review of the ability for variants of the Trail Making Test to assess cognitive impairment. Appl Neuropsychol Adult. 2022;29(6):1634-45.
31. Perianez JA, Lubrini G, Garcia-Gutierrez A, Rios-Lago M. Construct Validity of the Stroop Color-Word Test: Influence of Speed of Visual Search, Verbal Fluency, Working Memory, Cognitive Flexibility, and Conflict Monitoring. Arch Clin Neuropsychol. 2021;36(1):99-111.
32. Ricci M, Graef S, Blundo C, Miller LA. Using the Rey Auditory Verbal Learning Test (RAVLT) to differentiate alzheimer's dementia and behavioural variant fronto-temporal dementia. Clin Neuropsychol. 2012;26(6):926-41.
33. Arevalo-Rodriguez I, Smailagic N, Roque IFM, Ciapponi A, Sanchez-Perez E, Giannakou A, et al. Mini-Mental State Examination (MMSE) for the detection of Alzheimer's disease and other dementias in people with mild cognitive impairment (MCI). Cochrane Database Syst Rev. 2015;2015(3):CD010783.
34. Hobson J. The Montreal Cognitive Assessment (MoCA). Occup Med (Lond). 2015;65(9):764-5.
35. Seshadri M, Mazi-Kotwal N. A copyright-free alternative to the mini-mental state examination is needed. BMJ. 2012;345:e8589.
36. Safieh M, Korczyn AD, Michaelson DM. ApoE4: an emerging therapeutic target for Alzheimer's disease. BMC Med. 2019;17(1):64.
37. Colucci-D'Amato L, Speranza L, Volpicelli F. Neurotrophic Factor BDNF, Physiological Functions and Therapeutic Potential in Depression, Neurodegeneration and Brain Cancer. Int J Mol Sci. 2020;21(20).
38. Halfin A. Depression: the benefits of early and appropriate treatment. Am J Manag Care. 2007;13(4 Suppl):S92-7.
39. Stein MB, Hill LL. Are There Reasons to Fear Anxiety Screening? JAMA. 2023;329(24):2132-4.
40. Byrne GJ, Pachana NA. Anxiety and depression in the elderly: do we know any more? Curr Opin Psychiatry. 2010;23(6):504-9.
41. Smith PJ, Merwin RM. The Role of Exercise in Management of Mental Health Disorders: An Integrative Review. Annu Rev Med. 2021;72:45-62.
42. Scott AJ, Webb TL, Martyn-St James M, Rowse G, Weich S. Improving sleep quality leads to better mental health: A meta-analysis of randomised controlled trials. Sleep Med Rev. 2021;60:101556.
43. Fusar-Poli P, Salazar de Pablo G, De Micheli A, Nieman DH, Correll CU, Kessing LV, et al. What is good mental health? A scoping review. Eur Neuropsychopharmacol. 2020;31:33-46.

For peer review only - http://bmjopen.bmj.com/site/about/guidelines.xhtml

For peer review only

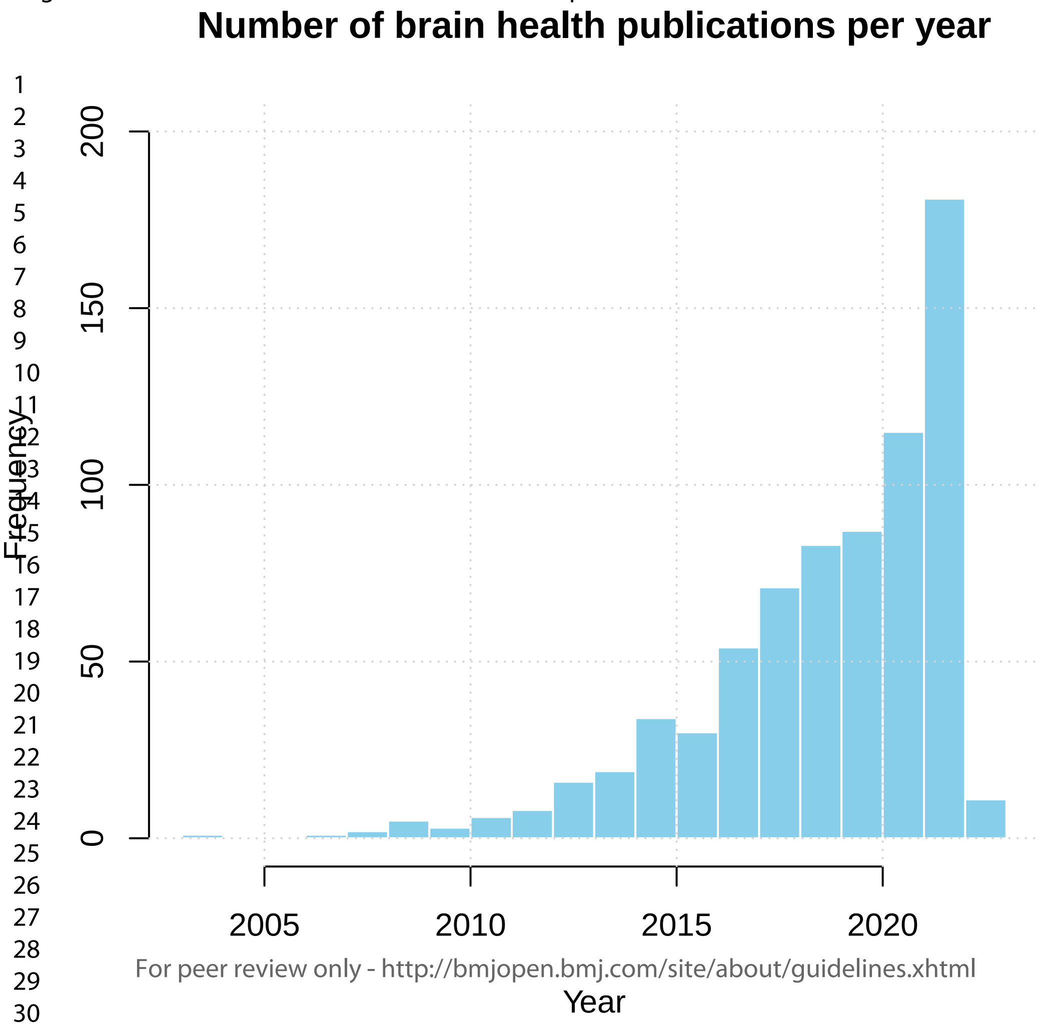
Number of brain health publications per year
Frequency
0
50
100
150
200
2005
2010
2015
2020
Year

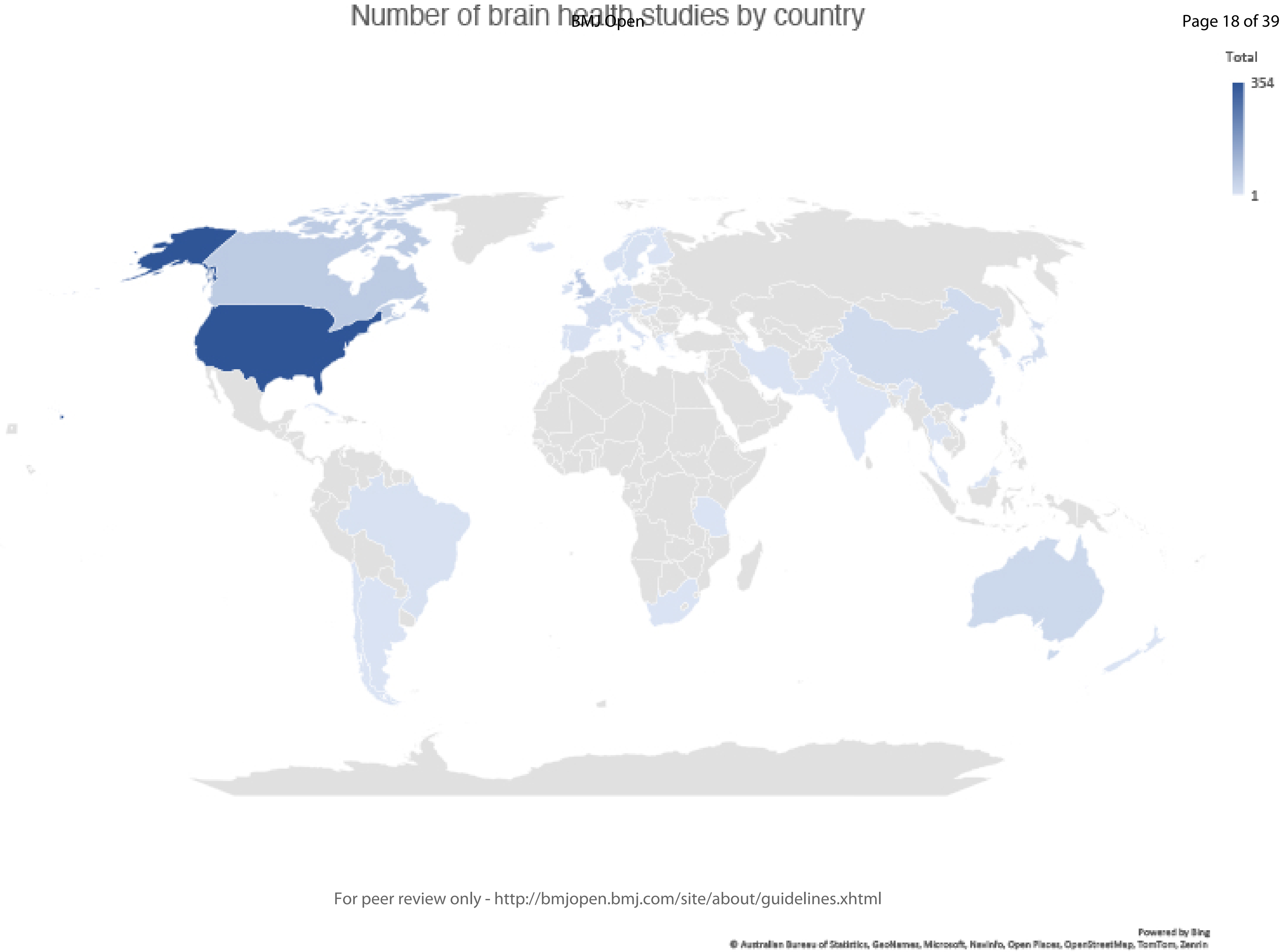
Number of brain health studies by country
Total
354
1
Powered by Bing
© Australian Bureau of Statistics, GeoNames, Microsoft, Navinfo, Open Places, OpenStreetMap, TomTom, Zenrin

Appendices and supplementary material

APPENDIX I: SEARCH STRATEGY

("brain health"(TextWord) AND Measurement terms (All Fields))
OR
((("Cerebrospinal fluid" OR "diagnostic imaging" OR anisotropy OR apolipoprotein OR apolipoproteins OR "arterial spin labelling" OR "brain derived neurotrophic factor" OR biomarker OR biomarkers OR "blood oxygen level dependent" OR "brain function" OR "brain imaging" OR "brain volume" OR "brain tissue volume" OR "cognitive assessment" OR "cognitive interview" OR "cognitive interviews" OR "DNA methylation" OR "genome wide association study" OR GWAS OR "gray matter" OR "grip strength" OR "hand strength" OR "hippocampal volume" OR "magnetic resonance imaging" OR MRI OR "magnetic resonance spectroscopy" OR "mental health statistics" OR "mental status and dementia tests" OR "mini mental state exam" OR "mini mental state examination" OR MMSE OR "motor control" OR "neurocognitive function" OR neuroimaging OR "neuropsychological tests" OR "neuropsychological testing" OR "non verbal reasoning" OR "nonverbal reasoning" OR "nutritional status" OR "physical function" OR "positron emission tomography" OR proteomic OR proteomics OR "psychological tests" OR "psychological testing" OR "self report" OR "short sleep duration" OR "surveys and questionnaires" OR "computed tomography" OR "voxel based morphometry" OR "white matter hyperintensities")) AND ((brain[Title] OR "white matter"[Title] OR Brain[MeSH Terms]) AND (atroph*[Title] OR Alzheimer*[Title] OR cogniti*[Title] OR dementia*[Title] OR "executive function"[Title] OR hippocamp*[Title] OR "mild cognitive impairment"[Title] OR memory[Title] OR "mental disorder"[Title] OR "mental health"[Title] OR neurocognitive[Title] OR neurodegeneration[Title] OR "postoperative cognitive"[Title] OR stroke*[Title] OR "vascular cognitive"[Title]) AND (health[Title] OR healthy[Title] OR longevity[Title] OR "protective factor*"[Title] OR "quality of life"[Title] OR psycholog*[Title] OR resilience[Title] OR well-being[Title] OR health[MeSH Terms] OR outcome assessment health care[MeSH Terms] OR quality of life[MeSH Terms] OR psychological well-being[MeSH Terms]))) OR (("brain health"[Text Word]) AND (("Cerebrospinal fluid" OR "diagnostic imaging" OR anisotropy OR apolipoprotein OR apolipoproteins OR "arterial spin labelling" OR "brain derived neurotrophic factor" OR biomarker OR biomarkers OR "blood oxygen level dependent" OR "brain function" OR "brain imaging" OR "brain volume" OR "brain tissue volume" OR "cognitive assessment" OR "cognitive interview" OR "cognitive interviews" OR "DNA methylation" OR "genome wide association study" OR GWAS OR "gray matter" OR "grip strength" OR "hand strength" OR "hippocampal volume" OR "magnetic resonance imaging" OR MRI OR "magnetic resonance spectroscopy" OR "mental health statistics" OR "mental status and dementia tests" OR "mini mental state exam" OR "mini mental state examination" OR MMSE OR "motor control" OR "neurocognitive function" OR neuroimaging OR "neuropsychological tests" OR "neuropsychological testing" OR "non verbal reasoning" OR "nonverbal reasoning" OR "nutritional status" OR "physical function" OR "positron emission tomography" OR proteomic OR proteomics OR "psychological tests" OR "psychological testing" OR "self report" OR "short sleep duration" OR "surveys and questionnaires" OR "computed tomography" OR "voxel based morphometry" OR "white matter hyperintensities")))

For peer review only - http://bmjopen.bmj.com/site/about/guidelines.xhtml

Search limits: English language, Human

For peer review only

For peer review only - http://bmjopen.bmj.com/site/about/guidelines.xhtml

APPENDIX II: PRISMA DIAGRAM

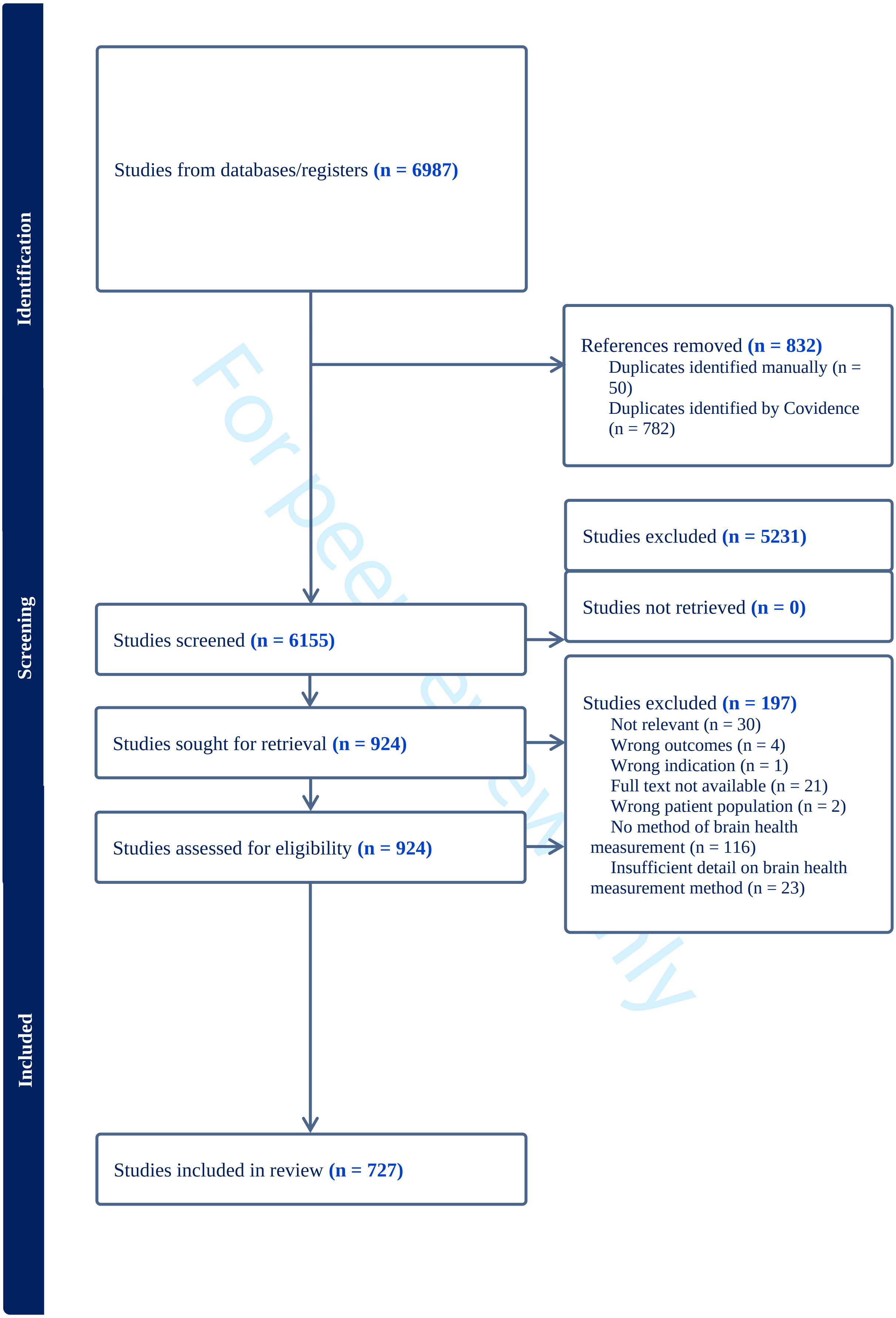

APPENDIX III: DATA EXTRACTION INSTRUMENT

All numbered items were presented as checkboxes for reviewers to tick for all that applied for each study.

Study characteristics

Author last name [whitespace]

Year of publication[whitespace]

Type of study

1. Randomised controlled trial
2. Cohort study
3. Systematic review/meta-analysis
4. Narrative review
5. Case series
6. Other

Any specific definition of brain health listed in paper? (optional) [whitespace]

Methods of brain health measurement

Imaging

Structural MRI

1. Total/whole brain volume
2. Total/whole brain gray matter volume
3. Total/whole brain white matter volume
4. Hippocampal volume
5. Gray matter volume in specific region(s)
6. White matter volume in specific region(s)
7. Cerebrospinal fluid (CSF) volume in specific region(s)
8. White matter hyperintensities (WMHs)
9. Cerebral microbleeds (CMBs)
10. Cortical thickness
11. White matter lesion volume

Functional MRI

1. Resting state functional connectivity
2. Task based functional connectivity
3. Cerebral blood flow (arterial spin labelling/BOLD responses)
4. Cerebral blood volume
5. Cerebral oxygen consumption
6. Cerebral metabolic rate of oxygen (CMRO2)

Diffusion MRI

For peer review only - http://bmjopen.bmj.com/site/about/guidelines.xhtml

1. Mean diffusivity
2. Axial diffusivity
3. Radial diffusivity
4. Fractional anisotropy
5. Fibre bundle lengths

Compound imaging

1. Brain age estimations (image based)
2. Brain age gap calculations
3. Brain atrophy and lesion index (BALI)
4. Brain health quotients (BHQ)

PET scans/ Spectroscopy/Misc

1. Amyloid load/status
2. Lactate
3. N-acetylaspartate
4. Glutamate
5. Glutamine
6. Magnetoencephalography (MEG) - functional connectivity

Other imaging parameters not previously specified[whitespace]

Genetics/blood/CSF/other

Genetics

1. ApoE4 allele/gene variation

Blood/CSF biomarkers

1. BDNF (serum/blood)
2. BDNF (CSF)
3. Tau (blood)
4. Tau (CSF)
5. Abeta42 or 40 (blood)
6. Abeta 42 or 40 (CSF)
7. IL8 (blood)
8. IL8 (CSF)
9. ACE angiotensin converting enzyme (blood)

EEG

1. Mindreader
2. EEG based brain age
3. EEG unspecified

Clinical

For peer review only

For peer review only - http://bmjopen.bmj.com/site/about/guidelines.xhtml

1. Hand grip strength
2. Clinical diagnosis of alzheimer's disease/dementia
3. Oculometric test
4. Duplex ultrasonography of carotids
5. Transcranial doppler ultrasound
6. Transcranial magnetic stimulation (TMS)

Mental health

1. Hospital anxiety and depression (HADS) scales
2. Centre for epidemiologic studies depression scale (CES-D)
3. Beck anxiety inventory
4. Beck depression inventory-ii
5. Pittsburgh Sleep Quality index
6. Mayo clinic fluctuations scale
7. Quality of life scale
8. Zung self rating depression scale
9. Self reported mental health history
10. Apathy scale

Combination scores

1. Brain health score (lifestyle based)
2. Brain health test (BHT)
3. Lifestyle for brain health (LIBRA) index
4. Resilience index

Any other clinical/mental health/combo scores/genetic measures not previously reported[whitespace]

Cognitive tests

Cognitive tests (A-Z)

1. Mini-mental status examination (MMSE)
2. Montreal cognitive assessment (MoCA)
3. Activities specific balance confidence scale
4. Adulthood cognitive activity questionnaire
5. Alzheimer's disease assessment cale (ADAS-cog)
6. Animal naming
7. Apathy inventory
8. Attention network test (ANT)
9. Auditory consonant trigrams
10. Awareness of social inference test (TASIT-R)
11. Behaviour rating inventory of executive function adult version (BRIEF-A)
12. Behavioural assessment of dysexecutive syndrome (BADS)
13. Bell cancellation test
14. Benson Figure Recall
15. Benton Visual Retention Test (BVRT)

For peer review only

16. Boston diagnostic aphasia exam
17. Boston naming test (BNT)
18. Brief cognitive ability measure (B-CAM)
19. Brief visuospatial memory test-revised (BVMT)
20. Buschke selective reminding test
21. California verbal learning test (CVLT-II)
22. Cambridge Cognition Paired Associates Learning
23. CANTAB intra extra dimensional set shift (IED)
24. Category fluency
25. CERAD word list delayed recall and memory battery
26. Clinical dementia rating (CDR)
27. Clock drawing test (CDT)
28. CNS vital signs
29. Cogniciti's brain health assessment (BHA)
30. Cogstate brief battery (CBB)
31. Cogstate neurocognitive battery
32. Cohen's relational memory
33. Color trails test
34. Color word interference test (CWIT)
35. COMPASS-ND neuropsychological battery
36. Continuous paired associative learning (CPAL) task
37. Controlled oral word association test
38. Corsi block test
39. Delis-Kaplan executive functioning (D-KEFS)
40. Digit span
41. Digit symbol substitution test (DSST)
42. Direct assessment of functional status revised (DAFS-R)
43. DKEFS Color Word Intereference
44. Edinburgh Handedness Inventory (EHI)
45. Everyday cognition scale (ECog)
46. Face name association test
47. Flanker NIH Toolbox
48. Frontal assessment battery (FAB)
49. Frontal systems behaviour scale (FrSBe)
50. Geriatric anxiety inventory
51. Gerontology functional assessment tool (NCGG-FAT)
52. Grooved pegboard (PEGS)
53. Hasegawa's dementia scale-revised (HDS-R)
54. Hooper visual organisation test
55. Hopkins verbal learning test (HVLT)
56. Identical pairs version
57. Illness intrusiveness rating scale (IIRS)
58. Inspection time task
59. Iowa Gambling Task
60. IQ test
61. Letter fluency
62. Letter number sequencing
63. Lifetime of experiences questionnaire (LEQ)
64. Logical memory

For peer review only

65. MATRICS consensus cognitive battery (MCCB)
66. Mattis dementia rating scale (DRS)
67. Maryo clinic study of ageinig (MCSA)
68. Memory tool box task
69. Memtrax memory test
70. MiniCog
71. Mnemonic similarity task (MST)
72. ModBent
73. [Modified] california verbal learning test
74. [Modified] Rey Auditory verbal learning test (RAVLT)
75. Moray House Test No.12
76. MSCEIT managing emotions
77. N- back working memory task
78. NAB mazes
79. National adult reading test
80. National institutes of health (NIH) Toolbox-cognitive battery
81. Neuropsychological assessment scales
82. Number letter computer task
83. Number symbol coding task
84. Pfeffer questionnaire
85. Philadelphia verbal learning test (PVLT)
86. Pittsburgh compound B
87. Positive and negative affect schedule (PANAS)
88. Preclinical Alzheimer's cognitive composite 5 (PACC5)
89. Reaction time tasks
90. Repeatable battery for the assessment of neuropsychological status (RBANS)
91. Rey-Osterrieth Complex Figure test (CFT)
92. RI-48 Cued recall test
93. Set switching
94. Shopping list memory task
95. Short portable mental status questionnaire (SPMSQ)
96. Spatial working memory
97. Stroop color-word test (SCWT)
98. Subjective cognitive decline-questionnaire (SCD-Q)
99. Sustained attention to response task (SART)
100. Symbol digit modalities test (SDMT)
101. TabCAT brain health assessment
102. Task switching tests
103. Technology activities of daily living questionnaire (T-ADLQ)
104. Telephone interview for cognitive status (TICS)
105. Test of everyday attention (TEA)
106. Test of variables of attention (TOVA)
107. The 2 and 7 test
108. Thurstone word fluency test (TWFT)
109. Trail making test (TMT) A or/and B
110. VCAP battery
111. Verbal fluency
112. WAIS-III letter number sequencing
113. Wechsler abbreviated scale of intelligence

For peer review only - http://bmjopen.bmj.com/site/about/guidelines.xhtml

114. Wechsler adult intelligence scale (revised)
115. Wechsler memory scale
116. Wechsler test of adult reading
117. Western aphasia battery revised (WAB-AQ)
118. Wide range achievement test (WRAT)
119. Wisconsin Card Sort Test

Additional cognitive tests not previously mentioned[whitespace]

For peer review only

Supplementary tables and figures

Table 1: Number of studies using different categories of brain health measurements*

Type of study	Number of studies	Imaging	Cognitive	Mental health	Clinical	Bio	>1 category (% of number of studies)
**Case series**	25	19	15	0	6	4	18 (72)
Cohort study	609	471	318	11	132	73	382 (63)
Randomised controlled trial	59	47	35	1	11	3	34 (58)
Narrative review	11	7	7	0	3	6	10 (91)
Systematic review/meta-analysis	19	15	12	1	8	7	16 (84)
Other	4	2	3	0	1	1	3 (75)

*Row data is not mutually exclusive as many studies used more than 1 category of methods

For peer review only

Table 2: Number of studies published by year using different categories of brain health measurements*

Year	No. of studies published	Imaging	Cognitive	Mental health	Clinical	Biological	No. of multicategory studies
**2003**	1	1	0	0	0	0	0
**2007**	1	1	1	0	0	0	1
**2008**	2	1	1	0	0	0	0
**2009**	5	4	4	0	2	1	3
**2010**	3	3	1	0	0	0	1
**2011**	6	5	4	0	4	2	2
**2012**	8	6	4	0	5	1	4
**2013**	16	14	10	0	2	5	6
**2014**	19	23	10	0	3	2	7
**2015**	34	33	16	0	4	5	16
**2016**	30	31	22	0	4	4	12
**2017**	54	53	32	2	6	6	22
**2018**	71	78	42	1	18	6	26
**2019**	83	84	63	2	22	6	41
**2020**	87	85	57	1	20	12	37
**2021**	115	115	62	2	30	10	44
**2022**	181	167	141	5	56	39	72
**2023**	11	8	4	0	3	3	11

*Row data is not mutually exclusive as many studies used more than 1 category of methods

For peer review only

Table 3: Number of brain health studies by country using different categories of brain health measurements. Row data is mutually exclusive.

Country	Imaging	Cognitive	Mental health	Clinical	Bio-logical	Multi-category	Number of studies (%)*
**United States**	93	24	0	8	6	221	354 (48.7)
**United Kingdom**	19	2	0	3	0	43	67 (9.2)
**Canada**	23	1	0	2	0	35	61 (8.4)
**Australia**	7	3	0	2	0	17	29 (4.0)
**China**#	6	2	0	2	0	12	22 (3.0)
**France**	3	1	0	0	0	14	18 (2.5)
**Japan**	5	2	0	1	0	7	15 (2.0)
**Netherlands**	1	2	0	0	0	11	14 (1.9)
**South Korea**	5	0	0	0	1	6	12 (1.7)
**Germany**	2	0	0	0	0	10	12 (1.7)
**Norway**	2	0	0	0	0	7	9 (1.2)
**Republic of Ireland**	1	1	0	0	0	7	9 (1.2)
**Spain**	2	2	0	1	1	2	8 (1.1)
**Brazil**	1	0	0	0	0	6	7 (1.0)
**Sweden**	1	0	0	0	0	6	7 (1.0)
**Taiwan**	1	0	0	0	0	5	6 (0.8)
**Czech Republic**	2	0	0	0	0	3	5 (0.7)
**Italy**	0	0	0	0	0	5	5 (0.7)
**Iran**#	1	1	0	0	0	2	4 (0.6)
**Hong Kong**	1	0	0	0	0	2	3 (0.4)
**Portugal**	1	0	0	0	0	2	3 (0.4)
**Switzerland**	1	1	0	0	0	1	3 (0.4)
**Belgium**	0	0	0	0	0	3	3 (0.4)
**Malaysia**#	2	0	0	0	0	0	2 (0.3)
**Argentina**#	1	0	0	0	0	1	2 (0.3)
**Israel**	1	0	0	1	0	0	2 (0.3)
**Finland**	0	0	0	1	0	1	2 (0.3)
**New Zealand**	0	1	0	0	0	1	2 (0.3)
**Cuba**#	1	0	0	0	0	0	1 (0.1)
**Pakistan**#	1	0	0	0	0	0	1 (0.1)
**Thailand**#	1	0	0	0	0	0	1 (0.1)
**India**#	0	0	0	0	0	1	1 (0.1)
**South Africa**#	0	1	0	0	0	0	1 (0.1)
**Tanzania**#	0	0	0	0	0	1	1 (0.1)
**Denmark**	1	0	0	0	0	0	1 (0.1)
**Greece**	1	0	0	0	0	0	1 (0.1)
**Hungary**	1	0	0	0	0	0	1 (0.1)
**Iceland**	1	0	0	0	0	0	1 (0.1)
**Chile**	0	0	0	0	0	1	1 (0.1)

*Only non-review articles included in this table
# denotes lower or middle income country as defined by the World Bank

For peer review only - http://bmjopen.bmj.com/site/about/guidelines.xhtml

Table 4: Structural MRI methods for brain health measurement*

Measurement method	Number of studies using this method (% of total studies)
Structural MRI	
Grey matter volume in specific region(s)	133 (18.3)
White matter hyperintensities (WMHs)	133 (18.3)
Total brain volume	132 (18.2)
Whole brain grey matter volume	106 (14.6)
Hippocampal volume	105 (14.4)
White matter volume in specific region(s)	95 (13.1)
Whole brain white matter volume	77 (10.6)
Cortical thickness	54 (7.4)
White matter lesion volume	29 (4.0)
Cerebrospinal fluid volume in specific region(s)	19 (2.6)
Cerebral microbleeds	16 (2.2)
Lacunes	4 (0.6)
Cortical superficial siderosis	2 (0.3)
Lacunar infarcts	2 (0.3)
Embolic infarcts	
Small vessel disease	2 (0.3)
Perivascular spaces	2 (0.3)
Functional MRI	
Cerebral blood flow in specific region(s)	51 (7.0)
Resting state functional connectivity	45 (6.2)
Task based functional connectivity	31 (4.3)
Cerebral metabolic rate of oxygen	2 (0.3)
Diffusion MRI	
Fractional anisotropy	102 (14.0)
Mean diffusivity	59 (8.1)
Axial diffusivity	19 (2.6)
Radial diffusivity	19 (2.6)
Free water	4 (0.6)
Fibre bundle lengths	3 (0.4)
Fibre density	3 (0.4)
Compound indices	
Brain age gap calculations	13 (1.8)
Brain atrophy and lesion index (BALI)	8 (1.1)
Brain age estimations (MRI based)	2 (0.3)
Brain health quotients (BHQ)	2 (0.3)
Spatial pattern of atrophy for recognition of brain aging (SPARE-BA)	2 (0.3)
Miscellaneous imaging	
PET amyloid load or presence	38 (5.2)
PET tau	11 (1.5)
Transcranial doppler ultrasound	9 (1.2)

For peer review only - http://bmjopen.bmj.com/site/about/guidelines.xhtml

Transcranial magnetic stimulation (TMS)	9 (1.2)
Functional near infrared spectroscopy (fNIRS)	8 (1.1)
PET FDG	7 (1.0)
Magnetoencephalography (MEG) – functional connectivity	7 (1.0)
MRS N-acetylaspartate	5 (0.7)
MRS glutamate	6 (0.8)
MRS glutamine	4 (0.6)
MRS lactate	4 (0.6)
Duplex ultrasonography of carotid arteries	4 (0.6)
PET florbetapir	3 (0.4)
Magnetic resonance elastography (MRE)	3 (0.4)
Pulsatility index	3 (0.4)
Iron content (quantitative susceptibility weighted MRI)	3 (0.4)
Myelin water fraction maps (MWF)	2 (0.3)

*Rows in the table are not mutually exclusive as many studies used more than one method of measurement.

For peer review only

Table 5: Cognitive tests for brain health measurement

Measurement method	Number of studies using this method (% of total studies)
Trail making test (TMT) A or/and B	86 (11.8)
Mini-mental status examination (MMSE)	73 (10.0)
Stroop test	64 (8.8)
Rey Auditory verbal learning test (RAVLT) (inc. modified)	50 (6.9)
Montreal cognitive assessment (MoCA)	48 (6.6)
Digit span	39 (5.4)
Digit symbol substitution test (DSST)	36 (5.0)
Verbal fluency	32 (4.4)
Wechsler adult intelligence scale (inc. modified)	32 (4.4)
Hopkins verbal learning test (HVLT)	24 (3.3)
Wechsler memory scale	22 (3.0)
California verbal learning test (CVLT-II)	20 (2.8)
Logical memory	19 (2.6)
Boston naming test (BNT)	17 (2.3)
Rey-Osterrieth Complex Figure test (CFT)	16 (2.2)
Symbol digit modalities test (SDMT)	15 (2.1)
TabCAT UCSF brain health assessment	15 (2.1)
Wechsler abbreviated scale of intelligence	15 (2.1)
Cogstate brief battery (CBB)	13 (1.8)
Controlled oral word association test	12 (1.7)
Delis-Kaplan executive functioning (D-KEFS)	12 (1.7)
Animal naming	11 (1.5)
Repeatable battery for the assessment of neuropsychological status (RBANS)	11 (1.5)
Clinical dementia rating (CDR)	10 (1.4)
CERAD word list delayed recall and memory battery	9 (1.2)
National adult reading test	9 (1.2)
Reaction time tasks	9 (1.2)
Everyday cognition scale (ECog)	8 (1.1)
Task switching tests	8 (1.1)
Benton Visual Retention Test (BVRT)	7 (1.0)
Grooved pegboard (PEGS)	7 (1.0)
N- back working memory task	7 (1.0)
National institutes of health (NIH) Toolbox-cognitive battery	7 (1.0)
Category fluency	6 (0.8)
Clock drawing test (CDT)	6 (0.8)
Spatial working memory	6 (0.8)
Wechsler test of adult reading	6 (0.8)
CANTAB intra extra dimensional set shift (IED)	5 (0.7)
CNS vital signs	5 (0.7)
Color trails test	5 (0.7)
Flanker NIH Toolbox	5 (0.7)
Hooper visual organisation test	5 (0.7)
Letter fluency	5 (0.7)
Letter number sequencing	5 (0.7)
Selective reminding test	5 (0.7)
Visual memory	5 (0.7)
Behaviour rating inventory of executive function adult version (BRIEF-A)	4 (0.6)
Brief visuospatial memory test (BVMT) (inc.modified)	4 (0.6)
DKEFS Color Word Interference	4 (0.6)

For peer review only - http://bmjopen.bmj.com/site/about/guidelines.xhtml

Global cognitive function (GCF) score	4 (0.6)
Mattis dementia rating scale (DRS)	4 (0.6)
Alzheimer’s disease assessment scale (ADAS-cog)	4 (0.6)
Paired associates’ task	4 (0.6)
Pattern comparison processing speed (PCPS)	4 (0.6)
Visual reproduction	4 (0.6)
Digit symbol coding	4 (0.6)
Brain health test (BHT)	4 (0.6)
Cogniciti's brain health assessment (BHA)	3 (0.4)
Bell cancellation test	3 (0.4)
Brief cognitive ability measure (B-CAM)	3 (0.4)
Cogstate neurocognitive battery	3 (0.4)
Edinburgh Handedness Inventory (EHI)	3 (0.4)
Eriksen-Flanker task	3 (0.4)
Face name association test	3 (0.4)
Memtrax memory test	3 (0.4)
MiniCog	3 (0.4)
Mnemonic similarity task (MST)	3 (0.4)
Preclinical Alzheimer's cognitive composite 5 (PACC5)	3 (0.4)
Telephone interview for cognitive status (TICS)	3 (0.4)
Test of variables of attention (TOVA)	3 (0.4)
Thurstone word fluency test (TWFT)	3 (0.4)
VCAP battery	3 (0.4)
Wide range achievement test (WRAT)	3 (0.4)
Wisconsin Card Sort Test	3 (0.4)
Dimensional change card sort (DCCS)	3 (0.4)
Four Choice reaction time	3 (0.4)
Go/No go	3 (0.4)
Oral reading recognition (ORR)	3 (0.4)
Pairs matching	3 (0.4)
Picture Sequence Memory (PSM)	3 (0.4)
Semantic fluency	3 (0.4)
Spatial reconstruction task	3 (0.4)
Study specific neuropsychological test battery (unnamed)	3 (0.4)
Working memory	3 (0.4)
Attention network test (ANT)	2 (0.3)
Auditory consonant trigrams	2 (0.3)
Cambridge Cognition Paired Associates Learning	2 (0.3)
Color word interference test (CWIT)	2 (0.3)
Continuous paired associative learning (CPAL) task	2 (0.3)
Corsi block test	2 (0.3)
Gerontology functional assessment tool (NCGG-FAT)	2 (0.3)
Hasegawa's dementia scale-revised (HDS-R)	2 (0.3)
Lifetime of experiences questionnaire (LEQ)	2 (0.3)
Mayo clinic study of aging (MCSA)	2 (0.3)
Positive and negative affect schedule (PANAS)	2 (0.3)
Sustained attention to response task (SART)	2 (0.3)
Western aphasia battery revised (WAB-AQ)	2 (0.3)
7-minute screening test	2 (0.3)
Addenbrooke's Cognitive Examination (ACE-III)	2 (0.3)
Benson Figure	2 (0.3)
Cambridge Neurological Test Automated Battery (CANTAB)	2 (0.3)
Card rotations test	2 (0.3)
Cognitive Activity questionnaire	2 (0.3)
Digit vigilance	2 (0.3)
Fluid intelligence	2 (0.3)
Forward and reverse memory span	2 (0.3)

For peer review only - http://bmjopen.bmj.com/site/about/guidelines.xhtml

Identical pictures test	2 (0.3)
Lifetime Experience Questionnaire	2 (0.3)
List sorting working memory (LSWM)	2 (0.3)
NCGG-FAT (National Center for Geriatrics and Gerontology Functional Assessment Tool)	2 (0.3)
Neuro-QoL	2 (0.3)
Paper folding task	2 (0.3)
Picture Vocabulary Test (PVT)	2 (0.3)
Preclinical Alzheimer's Cognitive score	2 (0.3)
Prospective memory	2 (0.3)
Story recall	2 (0.3)
Visual Inspection Time	2 (0.3)
Tower of London	2 (0.3)

*Rows in the table are not mutually exclusive as many studies used more than one method of measurement.

For peer review only

Table 6: Biological methods for brain health measurement

Measurement method	Number of studies using this method (% of total studies)
Blood ApoE4 genotype	41 (5.6)
Blood brain-derived neurotrophic factor (BDNF)	33 (4.5)
CSF neurofilament light	11 (1.5)
CSF tau levels	10 (1.4)
Blood Aß 42 or 40	9 (1.2)
Blood tau levels	7 (1.0)
Post-mortem neuropathological evaluation of brain tissue	4 (0.6)
Blood S100Beta levels	3 (0.4)
Blood vascular endothelial growth factor (VEGF)	3 (0.4)
CSF BDNF levels	2 (0.3)
Blood interleukin-8 (IL8) levels	2 (0.3)
Blood glial fibrillary acidic protein (GFAP) levels	2 (0.3)
Blood tumour necrosis factor alpha levels	2 (0.3)
Blood eotaxin levels	2 (0.3)
Blood macrophage inflammatory protein-1alpha levels	2 (0.3)

*Rows in the table are not mutually exclusive as many studies used more than one method of measurement.

For peer review only

Table 7: Clinical methods for brain health measurement

Measurement method	Number of studies using this method (% of total studies)
Electroencephalography (EEG)	24 (3.3)
Lifestyle for brain health (LIBRA) index	12 (1.7)
EEG based brain age	5 (0.7)
Mindreader (EEG software)	4 (0.6)
Clinical diagnosis of Alzheimer's disease or dementia	4 (0.6)
Hand grip strength	4 (0.6)
Pittsburgh Sleep Quality Index (PSQI)	3 (0.4)
RAND-36 health related quality of life survey	2 (0.3)
SF-36 (short form survey)	2 (0.3)
Test of premorbid functioning	2 (0.3)

*Rows in the table are not mutually exclusive as many studies used more than one method of measurement.

For peer review only

For peer review only - http://bmjopen.bmj.com/site/about/guidelines.xhtml

Table 8: Mental health methods for brain health measurement

Measurement method	Number of studies using this method (% of total studies)
Barratt impulsiveness scale	11 (1.5)
Geriatric depression scale	7 (1.0)
Patient health questionnaire (PHQ)-9 depression scale	5 (0.7)
Hospital anxiety and depression (HADS) scales	5 (0.7)
Beck depression inventory	5 (0.7)
Centre for epidemiologic studies depression scale (CES-D)	4 (0.6)
Perceived stress scale	3 (0.4)
PHQ-8 depression scale	3 (0.4)
Generalised anxiety disorder assessment (GAD)-7	3 (0.4)
Ruminative responses scale	2 (0.3)
Post-traumatic stress disorder checklist for DSM-V[#] (PCL-5)	2 (0.3)

*Rows in the table are not mutually exclusive as many studies used more than one method of measurement. #Diagnostic and Statistical Manual of Mental Disorders, Fifth Edition

For peer review only

For peer review only - http://bmjopen.bmj.com/site/about/guidelines.xhtml

Preferred Reporting Items for Systematic reviews and Meta-Analyses extension for Scoping Reviews (PRISMA-ScR) Checklist

SECTION	ITEM	PRISMA-ScR CHECKLIST ITEM	REPORTED ON PAGE #
**TITLE**			
Title	1	Identify the report as a scoping review.	
**ABSTRACT**			
Structured summary	2	Provide a structured summary that includes (as applicable): background, objectives, eligibility criteria, sources of evidence, charting methods, results, and conclusions that relate to the review questions and objectives.	
**INTRODUCTION**			
Rationale	3	Describe the rationale for the review in the context of what is already known. Explain why the review questions/objectives lend themselves to a scoping review approach.	
Objectives	4	Provide an explicit statement of the questions and objectives being addressed with reference to their key elements (e.g., population or participants, concepts, and context) or other relevant key elements used to conceptualize the review questions and/or objectives.	
**METHODS**			
Protocol and registration	5	Indicate whether a review protocol exists; state if and where it can be accessed (e.g., a Web address); and if available, provide registration information, including the registration number.	
Eligibility criteria	6	Specify characteristics of the sources of evidence used as eligibility criteria (e.g., years considered, language, and publication status), and provide a rationale.	
Information sources*	7	Describe all information sources in the search (e.g., databases with dates of coverage and contact with authors to identify additional sources), as well as the date the most recent search was executed.	
Search	8	Present the full electronic search strategy for at least 1 database, including any limits used, such that it could be repeated.	
Selection of sources of evidence†	9	State the process for selecting sources of evidence (i.e., screening and eligibility) included in the scoping review.	
Data charting process‡	10	Describe the methods of charting data from the included sources of evidence (e.g., calibrated forms or forms that have been tested by the team before their use, and whether data charting was done independently or in duplicate) and any processes for obtaining and confirming data from investigators.	
Data items	11	List and define all variables for which data were sought and any assumptions and simplifications made.	
Critical appraisal of individual sources of evidence§	12	If done, provide a rationale for conducting a critical appraisal of included sources of evidence; describe the methods used and how this information was used in any data synthesis (if appropriate).	
Synthesis of results	13	Describe the methods of handling and summarizing the data that were charted.	

For peer review only

SECTION	ITEM	PRISMA-ScR CHECKLIST ITEM	REPORTED ON PAGE #
**RESULTS**			
Selection of sources of evidence	14	Give numbers of sources of evidence screened, assessed for eligibility, and included in the review, with reasons for exclusions at each stage, ideally using a flow diagram.	
Characteristics of sources of evidence	15	For each source of evidence, present characteristics for which data were charted and provide the citations.	
Critical appraisal within sources of evidence	16	If done, present data on critical appraisal of included sources of evidence (see item 12).	
Results of individual sources of evidence	17	For each included source of evidence, present the relevant data that were charted that relate to the review questions and objectives.	
Synthesis of results	18	Summarize and/or present the charting results as they relate to the review questions and objectives.	
**DISCUSSION**			
Summary of evidence	19	Summarize the main results (including an overview of concepts, themes, and types of evidence available), link to the review questions and objectives, and consider the relevance to key groups.	
Limitations	20	Discuss the limitations of the scoping review process.	
Conclusions	21	Provide a general interpretation of the results with respect to the review questions and objectives, as well as potential implications and/or next steps.	
**FUNDING**			
Funding	22	Describe sources of funding for the included sources of evidence, as well as sources of funding for the scoping review. Describe the role of the funders of the scoping review.	

JBI = Joanna Briggs Institute; PRISMA-ScR = Preferred Reporting Items for Systematic reviews and Meta-Analyses extension for Scoping Reviews.

* Where *sources of evidence* (see second footnote) are compiled from, such as bibliographic databases, social media platforms, and Web sites.

† A more inclusive/heterogeneous term used to account for the different types of evidence or data sources (e.g., quantitative and/or qualitative research, expert opinion, and policy documents) that may be eligible in a scoping review as opposed to only studies. This is not to be confused with *information sources* (see first footnote).

‡ The frameworks by Arksey and O'Malley (6) and Levac and colleagues (7) and the JBI guidance (4, 5) refer to the process of data extraction in a scoping review as data charting.

§ The process of systematically examining research evidence to assess its validity, results, and relevance before using it to inform a decision. This term is used for items 12 and 19 instead of "risk of bias" (which is more applicable to systematic reviews of interventions) to include and acknowledge the various sources of evidence that may be used in a scoping review (e.g., quantitative and/or qualitative research, expert opinion, and policy document).

From: Tricco AC, Lillie E, Zarin W, O'Brien KK, Colquhoun H, Levac D, et al. PRISMA Extension for Scoping Reviews (PRISMAScR): Checklist and Explanation. Ann Intern Med. 2018;169:467–473. doi: 10.7326/M18-0850.

For peer review only
